# Genomic abnormalities of *TP53* define distinct risk groups of paediatric B-cell non-Hodgkin lymphoma

**DOI:** 10.1038/s41375-021-01444-6

**Published:** 2021-10-21

**Authors:** Alexander M. Newman, Masood Zaka, Peixun Zhou, Alex E. Blain, Amy Erhorn, Amy Barnard, Rachel E. Crossland, Sarah Wilkinson, Amir Enshaei, Julian De Zordi, Fiona Harding, Mary Taj, Katrina M. Wood, Despina Televantou, Suzanne D. Turner, G. A. Amos Burke, Christine J. Harrison, Simon Bomken, Chris M. Bacon, Vikki Rand

**Affiliations:** 1 School of Health & Life Sciences, Teesside University, Middlesbrough, UK; 2 National Horizons Centre, Teesside University, 38 John Dixon Lane, Darlington, UK; 3 Wolfson Childhood Cancer Research Centre, Translational and Clinical Research Institute, Newcastle University, Newcastle upon Tyne, UK; 4 Newcastle Genetics Laboratory, Newcastle upon Tyne Hospitals NHS Foundation Trust, Newcastle upon Tyne, UK; 5 Department of Paediatric Oncology, Royal Marsden Hospital, Sutton, Surrey UK; 6 Department of Cellular Pathology, Newcastle upon Tyne Hospitals NHS Foundation Trust, Newcastle upon Tyne, UK; 7 Division of Cellular and Molecular Pathology, Department of Pathology, University of Cambridge, Addenbrooke’s Hospital, Cambridge, UK; 8 CEITEC, Masaryk University, Brno, Czech Republic; 9 Department of Paediatric Haematology, Oncology and Palliative Care, Addenbrooke’s Hospital, Cambridge University Hospitals NHS Foundation Trust, Cambridge, UK; 10 Department of Paediatric and Adolescent Haematology and Oncology, The Great North Children’s Hospital, Newcastle upon Tyne Hospitals NHS Foundation Trust, Newcastle upon Tyne, UK

**Keywords:** Cancer genomics, B-cell lymphoma

## Abstract

Children with B-cell non-Hodgkin lymphoma (B-NHL) have an excellent chance of survival, however, current clinical risk stratification places as many as half of patients in a high-risk group receiving very intensive chemo-immunotherapy. *TP53* alterations are associated with adverse outcome in many malignancies; however, whilst common in paediatric B-NHL, their utility as a risk classifier is unknown. We evaluated the clinical significance of *TP53* abnormalities (mutations, deletion and/or copy number neutral loss of heterozygosity) in a large UK paediatric B-NHL cohort and determined their impact on survival. *TP53* abnormalities were present in 54.7% of cases and were independently associated with a significantly inferior survival compared to those without a *TP53* abnormality (PFS 70.0% vs 100%, *p* < 0.001, OS 78.0% vs 100%, *p* = 0.002). Moreover, amongst patients clinically defined as high-risk (stage III with high LDH or stage IV), those without a *TP53* abnormality have superior survival compared to those with *TP53* abnormalities (PFS 100% vs 55.6%, *p* = 0.005, OS 100% vs 66.7%, *p* = 0.019). Biallelic *TP53* abnormalities were either maintained from the presentation or acquired at progression in all paired diagnosis/progression Burkitt lymphoma cases. *TP53* abnormalities thus define clinical risk groups within paediatric B-NHL and offer a novel molecular risk stratifier, allowing more personalised treatment protocols.

## Introduction

Current treatments for high-grade paediatric B-cell non-Hodgkin lymphoma (B-NHL) in resource-rich countries are extremely effective, with over 93% of children being cured [1–5]. Following the recent demonstration of the benefit of the anti-CD20 monoclonal antibody rituximab, these same rates of cure are now achieved even in patients presenting with established high-risk clinical features (high lactate dehydrogenase (LDH), bone marrow (BM) and/or central nervous system (CNS) disease) [5]. This success, however, has required the use of intensive multi-agent chemo-immunotherapy regimens, associated with significant, predominantly acute, toxicity such as infection and mucositis as well as a small risk of long-term neurological side effects and second malignancy [6–8]. Accordingly, reduction in treatment intensity for at least some patients is a key objective. Unfortunately, previous attempts to reduce treatment intensity in high-risk bone marrow/CNS-positive patients resulted in unacceptable deterioration in survival, highlighting the need for biomarker-driven risk stratification to complement currently established clinical and laboratory risk features [2, 9].

In contrast to the excellent overall survival currently achieved, the outcome for children with primary refractory or relapsed B-NHL is extremely poor, with fewer than 30% successfully salvaged despite the routine use of high-dose chemotherapy and stem cell rescue [10–13]. Mounting toxicity in this heavily pre-treated group means that escalating intensity alone cannot provide the solution to improving outcome in these patients [14]. Instead, a more comprehensive understanding of the biological drivers of therapy resistance is essential to support the development of more effective and less toxic targeted therapies for this group of patients [15].

Genomic studies have greatly improved our understanding of the pathogenesis and clinically relevant heterogeneity of both Burkitt lymphoma (BL) and diffuse large B cell lymphoma (DLBCL) [16–28]. Amongst their findings is the frequent mutation in both diseases of the tumour suppressor gene *TP53*, inactivation of which has an established role in lymphomagenesis [29–33]. *TP53* mutation, often accompanied by loss of heterozygosity, is present in 25–50% of sporadic BLs and approximately 20% of adult DLBCLs at diagnosis, while a recent report suggested a lower incidence amongst paediatric large B-cell lymphomas [16–22, 24–28, 34–36]. Consistently, *TP53* mutations have been shown to be associated with adverse clinical outcomes in adult aggressive B-cell lymphoma, including DLBCL, and other lymphoid neoplasms [24, 34–38]. In chronic lymphocytic leukaemia (CLL), they are associated with a poor response to chemotherapy and detection of *TP53* mutation and 17p deletion is used to guide therapeutic decisions [39]. However, to date, no study has incorporated analysis of clinical outcome with a detailed characterisation of *TP53* alterations in paediatric B-NHL where BL predominates, and the potential utility of *TP53* status as a clinical risk stratifier in this age group has remained unclear.

To address this question, we collected and analysed a clinically annotated cohort of 95 paediatric B-NHL patients from the UK. Using sequencing and copy number (CN) microarray data we show that *TP53* abnormalities at presentation are associated with disease progression and poor outcome. Importantly, the absence of *TP53* abnormalities is associated with an extremely low risk of relapse even in high-risk patients defined by high tumour stage and LDH. Moreover, we demonstrate that biallelic *TP53* abnormalities are either maintained or acquired at the time of disease progression, implicating loss of *TP53* function in the development of treatment resistance. Finally, we show that *TP53* abnormalities are associated with complex chromosomal copy number profiles, identifying a potential mechanism underlying the evolution of the chemo-resistant disease.

## Methods

### Patients and clinical samples

B-NHL samples from UK hospitals registered with the Children’s Cancer and Leukaemia Group (CCLG) Tissue Bank between 1993 and 2014 were obtained following informed consent from participants or their parent/guardian. A minimum of three-years follow-up was obtained for all survivors. Lymphomas were re-classified according to the World Health Organisation (WHO) Classification of Tumours of Haematopoietic and Lymphoid Tissues [40]. *IG*-*MYC* status was confirmed by fluorescence in situ hybridisation (FISH) as described in Supplementary methods. The cohort comprised BL (*n* = 64), DLBCL (*n* = 19), Burkitt-like lymphoma with 11q aberration (BLL-11q, *n* = 5) and remaining cases which could not be fully classified (B-NHL, NOS *n* = 7).

### *TP53* mutation and 17p copy number analysis

*TP53* mutational status was assessed using whole-exome sequencing (WES, *n* = 90) or Sanger sequencing of exons 5 to 8 (*n* = 5). WES data were generated using Illumina Nextera Exome enrichment (*n* = 89) or TWIST Human Core Exome kit (*n* = 1) and sequenced on an Illumina NovaSeq within the Newcastle University Genomics Core Facility or Illumina HiSeq by Eurofins Genomics (Germany). Data were analysed using the Genome Analysis Toolkit (GATK 3.7) and variants called using Mutect2. PCR products for Sanger sequencing were amplified using primers designed for *TP53* (Supplementary Table 1) and sequenced by Eurofins Genomics. WES base calls were confirmed by Sanger sequencing in 39 cases, with 100% concordance between sequencing methods.

Copy number alterations (CNAs) of 17p and other chromosomes were identified using Affymetrix Cytoscan HD, Genome-wide Human SNP Array 6.0 or OncoScan arrays performed by Eurofins Genomics. Raw data were analysed and visualised in Nexus Copy Number 10.0 (BioDiscovery) to detect CNAs and copy number neutral loss of heterozygosity (CNN-LOH) in all samples.

#### Chromosomal complexity analysis

Complex patterns of chromosomal copy number abnormality were defined as those with a fluctuation between two or more copy number states involving two or more individual segments ≥100 kb on a chromosome arm. These included chromothripsis-like patterns of alternation between two copy number states as well as stepwise increase in copy number of chromosome arms. This definition excludes regions with heterozygous deletion followed by homozygous deletion, single regions of gain, deletion or CNN-LOH, and abnormalities affecting alternate chromosome arms.

#### Statistical analyses

Estimates of overall survival (OS) and progression-free survival (PFS) were calculated and compared using Kaplan–Meier methods, log-rank tests and Cox-regression models. OS was defined as the time from diagnosis to death from any cause, with censoring at the date of the last contact. PFS was defined as the date of diagnosis to the time of disease progression or death. We report three-year OS and PFS survival rates. All variables conformed to the proportional hazards assumption. Other comparisons were performed using Fisher’s exact test. Analyses were performed using R Bioconductor packages ‘survival’ for univariate and multivariate analysis and ‘survminer’ for visualisation of Kaplan–Meier survival curves.

## Results

### Patient demographics and clinical characteristics

The cohort consisted of the diagnostic tumour samples from 95 UK paediatric B-NHL patients. In total, 89 of the 95 (94%) cases were uniformly treated on FAB/LMB96 protocols (trial or interim guidelines) and had a complete follow-up with a median follow-up of 66.2 months (1–270.4 months) (Table 1). In keeping with previously published clinical trial cohorts [4, 41], the 89 FAB/LMB96-treated cases in the present study demonstrated a median age of 8 years, a male predominance of 3.4:1, a predominance (67%) of Burkitt lymphoma, a majority of high stage (III/IV) patients (73%), bone marrow disease in 18% and CNS disease in 6% of cases. *MYC* status was available for 84/89 samples (five cases failed FISH or had no available material), amongst which 63 (75%) had an *IG-MYC* translocation, including 56/58 BL, 4/16 DLBCL, 3/5 B-NHL, NOS and 0/5 BLL-11q cases. Survival estimates at three years for PFS and OS were 83.1% (95% CI 75.7–91.3) and 87.6% (95% CI 81.1–94.8), respectively. For those patients with disease progression (primary refractory or relapsed disease), the median time from the initial diagnosis to progression was 4.5 months (range 2.8–7.7 months). The additional 6 of the 95 cases (4 BL, 1 DLBCL and 1 B-NHL, NOS) were included in the genomic analysis only.

**Table 1:** Clinical and cytogenetic characteristics of the FAB/LMB96-treated paediatric B-NHL cases.

		FAB/LMB96-treated cohort
	Total cases	89
Diagnosis	BL	60 (67.4%)
	DLBCL	18 (20.2%)
	BLL-11q	5 (5.6%)
	B-NHL, NOS	6 (6.7%)
Median age at diagnosis (range), years		8 (0.5–17)
Sex	Male	68 (76.4%)
	Female	20 (22.5%)
	Not available	1 (1.1%)
Tumour stage	Stage I or II	24 (27.0%)
	Stage III or IV	65 (73.0%)
	Not available	0 (0.0%)
BM involvement	Y	16 (18.0%)
	N	71 (79.8%)
	Not available	2 (2.2%)
CNS involvement^a^	Y	5 (5.6%)
	N	83 (93.3%)
	Not available	1 (1.1%)
LDH > 2x ULN	Y	38 (42.7%)
	N	38 (42.7%)
	Not available	13 (14.6%)
*MYC* translocation	Y	63 (70.7%)
	N	21 (23.6%)
	Not available	5 (5.6%)
Risk Group	High	41 (46.1%)
	Intermediate	38 (42.7%)
	Low	4 (4.5%)
	Not available	6 (6.7%)
Treatment Group	Group A	4 (4.5%)
	Group B	68 (76.4%)
	Group C	15 (16.9%)
	Group unknown	2 (2.2%)
	Rituximab added^b^	2 (2.2%)
	No Rituximab	84 (94.4%)
	Rituximab unknown	3 (3.4%)
Outcome		
PFS	Progression/relapse	15 (16.9%)
	Median time to event (range), months	4.5 (0.9–7.7)
OS	Deaths	11 (12.4%)
	Median time to death (range), months	6.5 (0.9–11.1)

*BL* Burkitt lymphoma, *DLBCL* diffuse large B-cell lymphoma, *BLL-11q* Burkitt-like lymphoma with 11q aberrations, *B-NHL, NOS* B-cell non-Hodgkin lymphoma not otherwise specified, *Y* Yes, *N* No, *“-“* no event, hazard ratio not reported, *BM* bone marrow, *CNS* central nervous system, *CSF* cerebrospinal fluid, *LDH* lactate dehydrogenase, *ULN* upper limit of normal, *PFS* progression-free survival, *OS* overall survival.

^a^One case had CSF involvement.

^b^Two cases received rituximab from the start of first-line therapy, two other patients received rituximab following switch to group C therapy following post-CYM-1 reassessment.

### Genomic analysis of the *TP53* locus

**Fig. 1:**
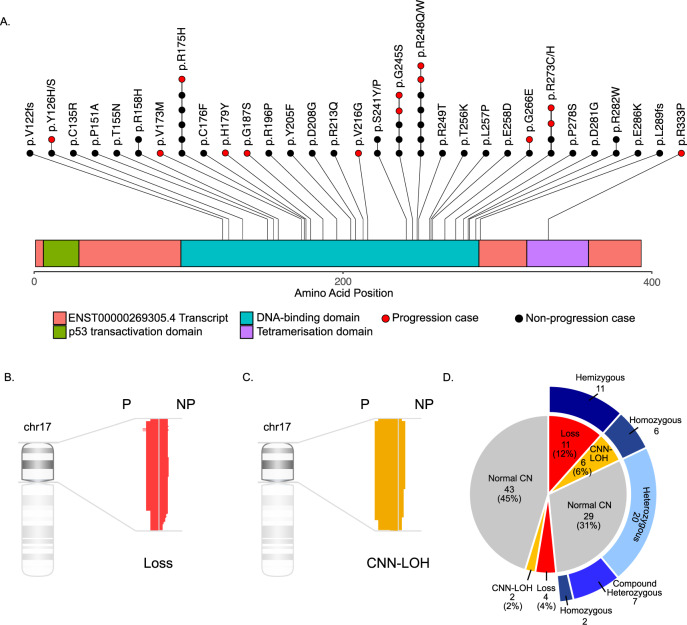
*TP53* mutations and genomic copy number abnormalities are detected in a high proportion of paediatric B-NHL cases. (**A**) A lolliplot showing 53 somatic and one germline mutation (R248Q detected in a Li-Fraumeni syndrome case). (**B**) Deletion of the *TP53* locus was detected in 3/14 patients with disease progression (P) and 12/78 with no disease progression (NP). (**C**) CNN-LOH of the *TP53* locus was detected in 5/14 patients with disease progression (P) and 3/78 with no disease progression (NP). (**D**) Representation of co-occurrence of *TP53* abnormalities. Inner circle represents *TP53* copy number status; outer ring represents *TP53* mutation status.

*TP53* mutations were found in 46/95 (48.4%) cases: 37 cases had a single non-synonymous somatic mutation, eight had two different somatic mutations and one had a germline mutation (DLBCL with Li-Fraumeni syndrome) (Fig. 1A, Supplementary Table 2). *TP53* mutations were found in 37/64 (57.8%) BL, 4/19 (21.0%) DLBCL, 4/7 (57.1%) B-NHL, NOS cases and 1/5 (20.0%) BLL-11q cases. All three DLBCL cases with somatic *TP53* mutations and all four mutated B-NHL, NOS cases carried *IG-MYC* rearrangements. The 35 distinct mutations included 33 missense mutations and two deletions leading to frameshifts. All but two mutations involved the DNA binding domain and experimental data from the UMD and IARC *TP53* databases showed all but two of the mutations to be functionally deleterious [42, 43]. As seen in other cancers [42, 44], the most commonly mutated residues were R175 (*n* = 6), G245 (*n* = 5), R248 (*n* = 7) and R273 (*n* = 4).

Next, we analysed 17p copy number alteration and identified deletions in 15/95 (15.8%) cases: 10/64 (15.6%) BL, 4/19 (21.0%) DLBCL and 1/7 (14.3%) B-NHL, NOS. The median region of deletion was 20.6 Mb (range 17.9–22.2 Mb), resulting in deletion of >80% of 17p (Fig. 1B). Additionally, 8/95 cases (8.4%) showed CNN-LOH: 7/64 (10.9%) BL and 1/19 (5.3%) DLBCL. The median region of CNN-LOH was 19.5 Mb (range 12.9–22.3 Mb), again covering >80% of 17p (Fig. 1C). All 17p deletions and CNN-LOH involved the *TP53* locus. The presence or absence of *TP53* copy number deletion was assessed by FISH in 76/95 cases: 49/64 BL, 16/19 DLBCL, 5/5 BLL-11q and 6/7 B-NHL, NOS (Supplementary Table 2). These included 12/15 cases with *TP53* deletion, 5/8 with CNN-LOH and 59/72 with neither. In each case, the FISH results were concordant with the copy number array findings.

Combining the mutation and CN data for the *TP53* locus showed that abnormalities are common at presentation with 52/95 (54.7%) cases harbouring at least one abnormality and functionally inactivating biallelic events present in 26/95 (27.4%) cases (Fig. 1D; Supplementary Table 2). Of the 26 cases with monoallelic *TP53* abnormalities, 20 had a single somatic heterozygous mutation (median variant allele frequency 39%, range 18–50%), four had a deletion and two had CNN-LOH. Among the 26 cases with biallelic abnormalities, 17 had *TP53* mutation together with a deletion or CNN-LOH, two had a homozygous mutation and seven had compound heterozygous mutations. The correlation between p53 protein expression and *TP53* status was assessed by p53 immunohistochemistry (IHC) in a subset of cases. In most cases, *TP53* mutation was associated with overexpression of p53 protein but the correlation between p53 expression levels and *TP53* status was variable and IHC could not reliably detect the range of genomic alterations (Supplementary Table 3).

### *TP53* abnormalities are associated with chromosomal complexity

**Fig. 2:**
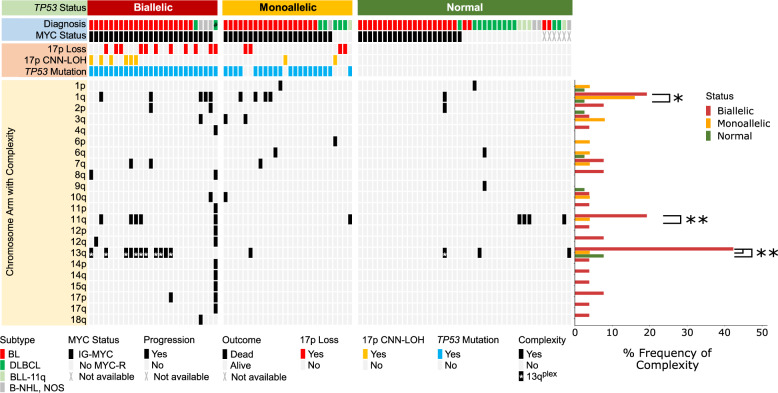
A pattern of complex chromosomal abnormalities in paediatric B-NHL is associated with *TP53* status. Oncoplot showing *TP53* status (upper panel) and associated complex chromosomes (lower panel). Histogram displaying the frequency of complex abnormalities of each chromosome arm in biallelic, monoallelic and *TP53* normal groups. As a diagnosis of BLL-11q is determined by the presence of a complex 11q rearrangement, these five cases were excluded from this analysis. *TP53* Biallelic with 1q complexity vs *TP53* Normal with 1q complexity, *p* = 0.026; *TP53* Biallelic with 11q complexity vs *TP53* Normal with 11q complexity, *p* = 0.006. *TP53* Biallelic with 13q complexity vs *TP53* Normal with 13q complexity, *p* = 0.001; *TP53* Biallelic with 13q complexity vs *TP53* Monoallelic with 13q complexity, *p* = 0.002. Fisher’s Exact test * = *p* < 0.05, ** = *p* < 0.01; ^#^ Li-Fraumeni syndrome case.

Genomic *TP53* alterations have been associated with genomic complexity in DLBCL and other tumours [21, 33, 44–46]. In this study, *TP53* abnormalities were not associated with the number of CNAs or the percentage of genome altered (Supplementary Table 4). However, *TP53* abnormalities were associated with complex CN profiles of specific chromosomes. (Fig. 2; Supplementary Table 5). Excluding BLL-11q, which is defined by a complex 11q rearrangement, chromosomes with complex CNAs were present in 30/51 cases with *TP53* abnormality but only in 5/39 cases with wild-type *TP53* (*p* < 0.001). This association was particularly strong for BL, in which 24/40 (60%) *TP53* abnormal cases harboured a complex chromosome compared to only 1/24 (4.2%) *TP53* wild-type cases (*p* = 0.001). The Li-Fraumeni syndrome case (DLBCL with *TP53* biallelic abnormality) demonstrated the highest number of complex CNAs within the whole cohort, but otherwise there was no association between *TP53* abnormalities and complex CNAs in DLBCL cases.

Most notably, complex CNAs of 1q, 11q and 13q were associated with biallelic *TP53* abnormalities (*p* < 0.05) (Fig. 2). Complexity of 1q typically involved stepwise increases in copy number, with only two cases having telomeric deletion (Supplementary Fig. 1). Both chromosome arms 11q and 13q recurrently showed a pattern of a region of gain adjacent to telomeric deletion, similar to the profile defining BLL-11q cases [47] (Supplementary Figs. 2–3). All ten cases with this characteristic gain-telomeric deletion pattern of 13q (here termed 13q^plex^) were BL (Supplementary Table 5). The *MIR17HG* locus mapped within the region of 13q CN gain/amplification, notably located in the highest peak for 9/10 (90%) 13q^plex^ cases (Supplementary Fig. 3).

### Survival analysis of *TP53* abnormalities in paediatric B-NHL

The clinical characteristics of cases with or without a *TP53* abnormality are presented in Supplementary Table 6. Univariate survival analyses identified a significantly adverse PFS for patients with high disease stage, bone marrow involvement or high LDH (Supplementary Table 7). CNS involvement was associated with a hazard ratio of 3.0 but did not reach significance, likely due to the low number of cases (*n* = 5). Those with mutations had significantly inferior three-year survival compared to those without (PFS 66.7% (95% CI 54.2–82.0) vs 100% (95% CI 100–100), *p* < 0.001 and OS 75.6% (95% CI 64.0–89.2) vs 100% (95% CI 100–100), *p* < 0.001) (Supplementary Fig. 4A, B), although no hazard ratio could be reported due to the absence of events in cases without *TP53* mutation. Likewise, 17p CNN-LOH involving *TP53* was also associated with worse outcome (PFS HR 4.9 (95% CI 1.6–15.6), *p* = 0.007; OS HR 4.7 (95% CI 1.2–17.7), *p* = 0.023) (Supplementary Fig. 4C, D, Supplementary Table 7). In contrast, as previously reported in the context of the FAB/LMB96 trial, univariate analysis showed that, agnostic of *TP53* mutation status, cases with deletion of 17p including the *TP53* locus did not have inferior outcome (Supplementary Fig. 4E, F, Supplementary Table 7) [2].

**Fig. 3:**
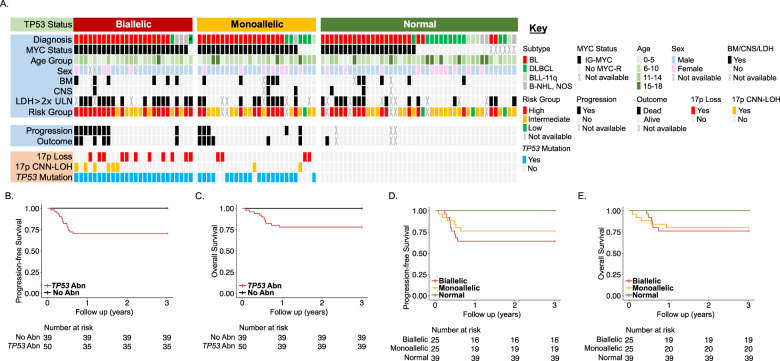
Identification of patient risk groups based on *TP53* status. (**A**) Oncoplot showing *TP53* status with clinical and molecular parameters as described in the key. Data is plotted from left to right according to *TP53* status. Kaplan–Meier plots showing (**B**) progression-free and (**C**) overall survival for any *TP53* abnormality (deletion, CNN-LOH and/or mutation), and (**D**) progression-free and (**E**) overall survival according to normal (green), monoallelic (amber) or biallelic (red) *TP53* status. # Li-Fraumeni syndrome case.

Combining the *TP53* locus alterations (Fig. 3A), patients with at least one *TP53* abnormality had a significantly inferior 3-year survival compared to those with no abnormality (PFS 70.0% (95% CI 58.4–83.9) vs 100 (95% CI 100–100), *p* < 0.001 and OS 78.0% (95% CI 67.3–90.4) vs 100 (95% CI 100–100), *p* = 0.002) (Fig. 3B, C). Importantly, those patients without any *TP53* abnormality at initial diagnosis had a PFS of 100% (95% CI 100–100) and an OS of 100% (95% CI 100–100). Most *TP53* abnormalities were seen in patients diagnosed with BL and the presence of any *TP53* abnormality remained adversely prognostic within this subgroup (PFS 67.5% (95% CI 54.4–83.7) vs 100% (95% CI 100–100), *p* = 0.005 and OS: 75% (95% CI 62.7–89.7) vs 100 (95% CI 100–100, *p* = 0.017) (Supplementary Fig. 5). Both monoallelic and biallelic *TP53* abnormalities were associated with adverse outcome, when compared with *TP53* wild-type cases (monoallelic PFS 76.0% (95% CI 61.0–94.7) vs 100% (95% CI 100–100), *p* = 0.001 and OS 80.0% (95% CI 65.8–97.3) vs 100% (95% CI 100–100), *p* = 0.004*;* biallelic PFS 64.0% (95% CI 47.7–85.9) vs 100% (95% CI 100–100), *p* < 0.001 and OS 76.0% (95% CI 61.0–94.7) vs 100 (95% CI 100–100) *p* = 0.001), but with no significant difference identified between these two groups (Fig. 3D, E). There was no association between prognosis and either complex CNA patterns of 1q, 11q or 13q (Supplementary Table 8) or gain of *MIR17HG*. Neither was there a difference in outcome for patients with or without any complex CNA (PFS HR 1.7 (95% CI 0.6–4.7), *p* = 0.295; OS HR 1.8 (95% CI 0.5–5.9), *p* = 0.332).

Multivariate analysis was performed to assess the impact of *TP53* abnormalities alongside established high-risk clinical factors (BM and CNS involvement, stage and high LDH) (Supplementary Table 7). Due to the lack of an event amongst the *TP53* wild-type patients included in this analysis, we are unable to report a hazard ratio for *TP53* status; however, having included this strong prognostic factor in the model, those established high-risk clinical factors were not independently significant.

### *TP53* status risk stratifies patients with clinically defined high-risk disease

**Fig. 4:**
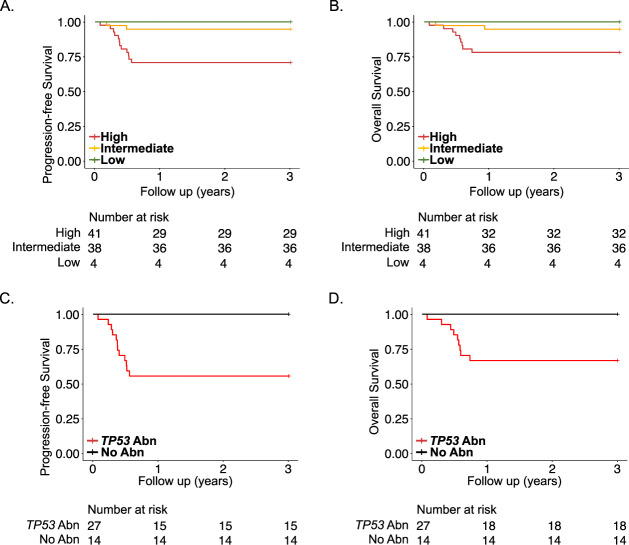
*TP53* status differentiates patients in the clinically defined high-risk group in paediatric B-NHL. Kaplan–Meier plots showing (**A**) progression-free and (**B**) overall survival for high, intermediate and low clinical risk groups, and (**C**) progression-free and (**D**) overall survival in high-risk patients according to the presence of any *TP53* abnormality.

The Inter-B-NHL ritux 2010 trial (NCT01516580) has recently demonstrated the survival benefit of adding rituximab to modified FAB/LMB96 chemotherapy for patients with high-risk disease (stage III and LDH > 2x ULN, stage IV or Burkitt leukaemia) [5]. Nevertheless, rituximab-chemotherapy was associated with increased incidence of prolonged hypogammaglobulinaemia and may be associated with a greater number of infections [5]. Given the inferior outcome of these patients prior to the use of rituximab and the unacceptable deterioration in that outcome with a previous attempt to reduce therapy intensity [9], they represent a key group for further biomarker-driven stratification. Therefore, we sought to understand the prognostic impact of *TP53* abnormalities specifically in this group. High-risk patients had a higher rate of *TP53* abnormality (27/41, 66%) than low/intermediate-risk groups (20/42, 48%), but here this association was specifically within the *TP53* biallelic group (*p* = 0.037) (Supplementary Table 6). As expected, the high-risk group had an inferior survival compared to the intermediate and low-risk groups (PFS 70.7% (95% CI 58.1–86.1) vs 94.7% (95% CI 87.9–100.0) vs 100.0% (95% CI 100.0–100.0), *p* = 0.013) and OS 78.6% (95% CI 66.4–91.8) vs 94.7% (95% CI 87.9–100.0) vs 100.0% (95% CI 100.0–100.0), *p* = 0.067) (Fig. 4A, B). Strikingly, despite the substantial clinical value of stage and LDH, the absence of any *TP53* abnormality in high-risk patients remained strongly associated with an excellent clinical outcome compared to high-risk patients with any *TP53* abnormality (PFS 100% (95% CI 100–100) vs 55.6% (95% CI 39.7–77.9), *p* = 0.005 and OS 100% (95% CI 100–100) vs 66.7% (95% CI 51.1–87.0), *p* = 0.019) (Fig. 4C, D). Stratification of the low/intermediate-risk group identified an inferior outcome for patients with any *TP53* abnormality but this did not reach significance (PFS 100 % (95% CI 100–100) vs 90% (95% CI 77.8–100%) vs, *p* = 0.133 and OS 100% (95% CI 100–100) vs 90% (95% CI 70.7–100%), *p* = 0.133) (Supplementary Fig. 6).

### Biallelic *TP53* abnormalities evolve during disease progression

**Fig. 5:**
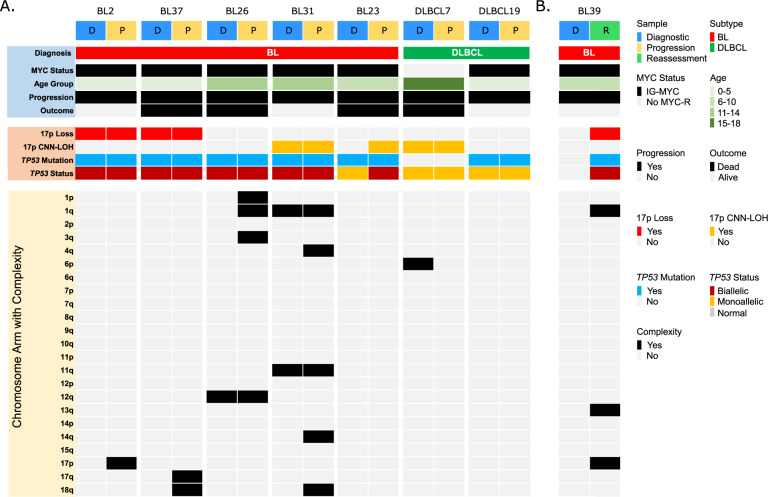
Biallelic *TP53* abnormalities are either maintained or acquired at the time of progression of BL. (**A**) Overview of clinical and molecular parameters for seven patients with matched samples taken at initial diagnosis (D) and at the time of progression (P). (**B**) One patient had matched samples from initial diagnosis and the time of reassessment.

Very little is known about the genomic changes associated with therapy resistance and disease progression in BL or other paediatric B-NHL [48]. To understand the evolution of *TP53* abnormalities associated with therapy resistance we investigated the paired biopsies taken at the time of disease progression for 7 cases and one further paired biopsy (patient BL39) taken from a viable residual tumour mass detected at routine reassessment following the CYM-1 component of FAB/LMB96 therapy. Amongst six BL cases, four had major clonal biallelic *TP53* abnormalities at the time of the original diagnosis (Fig. 5 and Supplementary Table 9) and these same biallelic abnormalities were maintained at the time of disease progression. Interestingly, the other two BL cases showed acquisition of biallelic *TP53* abnormalities in the second biopsies, further supporting a role for *TP53* in treatment resistance. The first, BL23, had only a monoallelic clonal G245S mutation at diagnosis but developed CNN-LOH at relapse, rendering this mutation biallelic. The other, BL39, lacked a detectable *TP53* abnormality at initial diagnosis but the residual tumour had both a deletion of chromosome 17p and a classical pathogenic R248W mutation. Analysis of WES data failed to identify even a minor clonal R248W mutation at presentation (0/124 reads). In contrast to the BL cases, both DLBCL cases with paired material harboured a monoallelic *TP53* abnormality at both diagnosis and progression. In five of the six BL cases, the therapy-resistant disease was associated with an increase in the number of chromosomes showing a complex copy number profile (Fig. 5).

## Discussion

The addition of rituximab to first-line therapy for children with high-risk B-NHL has resulted in ≥93% event-free survival (EFS) in all risk groups [5]. This success requires intensive multi-agent chemotherapy and comes at the cost of significant acute toxicity and there now exists a growing understanding of the long-term sequelae [6–8]. However, with very few relapse events occurring, identifying the prognostic biomarkers required for improved risk stratification in this rare patient group has become extremely challenging. Consequently, we undertook an analysis of the prognostic significance of *TP53* status in a large national cohort of paediatric mature B-NHL cases diagnosed prior to the routine introduction of rituximab therapy. This showed that the presence or absence of *TP53* abnormalities defines two patient groups with markedly different progression-free and overall survival rates, adverse and favourable respectively.

The principal clinical challenges in paediatric B-NHL have, for some years, been: (1) improving survival in high-risk patients; (2) reducing therapy intensity without increasing risk of relapse [9]; (3) identifying mechanisms of therapy resistance which result in extremely poor survival following relapse. Addressing the first of these challenges, the international collaborative trial Inter-B-NHL ritux 2010 (NCT01516580) recently demonstrated an 11% increase in EFS in high-risk patients (defined as stage III with LDH greater than twice the upper limit of normal, stage IV or Burkitt leukaemia) with the addition of six doses of rituximab to a modified FAB/LMB96 chemotherapy schedule [5]. This contrasts with the unacceptable reduction in survival seen in high-risk bone marrow/central nervous system positive patients randomised to reduced intensity therapy within the FAB/LMB96 study [9]. Nevertheless, within the reduced intensity arm of the high-risk FAB/LMB96 study, an 80% EFS was seen, implying that only a minority of patients benefit from intensification of treatment. The inability to identify those with a low risk of treatment failure has hampered risk stratification beyond clinicopathological features. Here, we show that within the high-risk patient group, (as defined in the Inter-B-NHL trial), *TP53* wild-type patients have a very low risk of relapse, despite the absence of rituximab therapy. If validated in international trial cohorts, analysis of *TP53* status may allow identification of a subset of patients currently considered high-risk for whom chemo-immunotherapy can be de-intensified without compromising efficacy. Initially, this could involve omitting rituximab but further reduction in chemotherapy similar to that attempted within FAB/LMB96, could also be considered. Our finding provides a potential biomarker platform for future trials of therapy reduction, albeit that a large number of patients and a more effective salvage strategy would be necessary for such a trial.

Critical to the better treatment of progressive or relapsed disease is a much deeper understanding of the drivers of therapy resistance. The finding that *TP53* abnormalities are associated with increased risk of disease progression, and our analysis of paired Burkitt lymphoma samples showing that all cases either maintained or developed biallelic *TP53* abnormalities at progression, suggest a key role for *TP53* loss of function in this process. In support of this assertion, Reutter et al. [49] found multiple *TP53* abnormalities at diagnosis and relapse in each of the five relapsed BL cases studied. Given the many biological pathways impacted by p53, the mechanisms by which *TP53* abnormalities promote therapy resistance merits further investigation in support of developing an effective salvage therapy. It is notable, therefore, that we found a strong association between *TP53* abnormalities and *MYC* rearrangements in our cohort. Since these two cancer genes cooperate to drive experimental lymphomagenesis in mice and have been associated with a particularly poor prognosis when concurrently mutated and translocated, respectively, in adult DLBCL, a deeper mechanistic understanding of the interactions between p53 and MYC in paediatric B-NHL may be particularly informative in this regard [29, 32, 38].

Mutations, present in half of cases, were the most frequent *TP53* abnormalities detected in our cohort. As expected, *TP53* deletions and CNN-LOH were less prevalent and were mostly present in tumours with *TP53* mutation [2, 50–53]. Overall, half of all cases with a *TP53* abnormality had biallelic alterations expected to abrogate wild-type *TP53* functions at diagnosis and biallelic events were present in all BL tumours at the time of progression. However, while the presence of any *TP53* abnormality was associated with an increased risk of disease progression and death, there was no difference in PFS or OS between cases with monoallelic or biallelic alterations at diagnosis. This contrasts with recent findings in myelodysplastic syndrome and plasma cell myeloma, in which specifically biallelic *TP53* alterations at diagnosis are associated with poor survival [54–56]. The increasing evidence that many mutant p53 proteins exhibit gain of function or dominant-negative properties may provide a partial explanation but our analysis of paired diagnosis/progression BL samples suggests the alternative explanation that clones/subclones with monoallelic *TP53* abnormalities may evolve to a biallelic state during disease progression under the selective pressure of therapy [32, 33, 57]. Interesting in this regard is the recent report of a case of relapsed BL with the expansion of a low level (2% VAF) *TP53* R248Q mutation at diagnosis to a 93% VAF at relapse, secondary to a combination of clonal expansion and CNN-LOH [58].

*TP53* mutations are associated with several types of genomic instability, including aneuploidy and chromothripsis, and thus increased genome complexity across the cancer spectrum [33, 44, 46]. Notably, a recently described subset of adult DLBCL carrying frequent biallelic inactivation of *TP53* by mutation and 17p deletion is selectively associated with an increase in small and large CNAs [21, 45]. Similarly, an association between *TP53* abnormalities and chromothripsis-like changes, including of 13q, has been reported in adult DLBCL [59, 60]. Although we did not see an effect on global genomic complexity in our predominantly BL paediatric cohort, we did observe the correlation of *TP53* abnormalities with complex copy number patterns involving 1q, 11q and 13q. The 13q gains centred on the *MIR17HG* gene, in keeping with a previous report of an association between 17p deletion and *MIR17HG* gain in a small sample set [50]. That report speculated that *MIR17HG* gain was associated with relapse but our data suggest that this probably results indirectly from the association of *MIR17HG* gain with *TP53* abnormalities [50].

We have demonstrated the clinical importance of *TP53* abnormalities in paediatric B-NHL, identifying them as a potential biomarker capable of further risk-stratifying patients currently considered high-risk. These findings now need to be validated in a large international trial cohort. For those children without a *TP53* abnormality, the risk of disease progression is extremely low and stratified trials examining therapy reduction should be considered. Evolution of biallelic abnormalities at relapse implicates *TP53* biology as a driver of therapy resistance and relapse and further understanding of the underlying mechanisms could lead to new strategies to prevent or treat therapy-resistant disease.

## Supplementary information

Supplemental Methods

Supplemental Tables

Supplemental Figure Legends

Supplemental Figure 1

Supplemental Figure 2

Supplemental Figure 3

Supplemental Figure 4

Supplemental Figure 5

Supplemental Figure 6
