# Supplementary material for: Genomic abnormalities of TP53 define distinct risk groups of paediatric B-cell non-Hodgkin lymphoma
Source: Leukemia. 2021 Oct 21;36(3):781–9. doi: 10.1038/s41375-021-01444-6 (PMC8885412; doi:10.1038/s41375-021-01444-6)
Supplement: Supplementary file 1 — Supplemental Methods [file 41375_2021_1444_MOESM1_ESM.docx]

**Supplemental methods**

**Supplemental Methods**

*Patients and Clinical Samples*

Project and ethics approval were granted via the CCLG Biological Studies Steering Group (REC 18/EM/0134; Biological Study 2012 BS08). The samples were either frozen or formalin-fixed and paraffin embedded. In total, 95 diagnostic tumour samples (41 with matched constitutional DNA), 7 matched tumour samples taken at the time of disease progression (primary refractory or relapsed disease), and one matched reassessment biopsy taken post-CYM1 were included. The inclusion criteria for the study were: (i) an initial diagnosis of Burkitt lymphoma, diffuse large B-cell lymphoma or related B-NHL subtype (e.g. Burkitt-like); (ii) fresh frozen or FFPE material banked with consent for research purposes with sufficient material to analyse copy number and mutation status of *TP53* and (iii) sufficient material and/or diagnostic information for pathology review. No cases meeting these criteria were excluded.

*Genomic copy number data analysis*

DNA was extracted using the Qiagen AllPrep DNA/RNA Mini kits for fresh frozen (FF) or formalin-fixed paraffin-embedded (FFPE) samples and quantified using the Qubit assay (ThermoFisher Scientific). DNA was hybridised to either the Affymetrix Cytoscan HD, Genome-wide Human SNP Array 6.0 array or OncoScan arrays by AROS Applied Biotechnology (Denmark) according to manufacturers’ protocols. For Affymetrix OncoScan arrays, the OSCHP files were generated from the array fluorescence intensity data (.CEL files) using OncoScan Console software. Copy number analysis was performed using Nexus Copy Number 10.0 (BioDiscovery). Both Cytoscan HD and SNP 6.0 arrays were analysed from raw .CEL files using the SNPRank algorithm, which is based on Circular Binary Segmentation, using 0.15 and -0.15 as gain and loss thresholds respectively with a minimum of 10 probes per segment called and a maximum contiguous probe spacing of 1Mb. Genome complexity was investigated by calculating the number of copy number segments called for each sample and the percentage of the total genome altered. All regions detected were subjected to rigorous quality control, including checks for known CNVs and low probe coverage, and confirmed by three independent researchers (VR, PZ and AN). The human reference genome was GRCh37/hg19.

*Whole-exome sequencing*

Whole-exome sequencing data were generated using Illumina Nextera Exome enrichment (n=89) or TWIST Human Core Exome kit (n=1) and sequenced on an Illumina NovaSeq within the Newcastle University Genomics Core Facility or Illumina HiSeq by Eurofins Genomics (Germany). Data was analyzed using Genome Analysis Toolkit (GATK 3.7)^1, 2^ and raw FASTQ files were aligned to human reference genome 37 decoy version aligned using BWA-MEM. Aligned SAM files were sorted, converted to BAM, indexed and PCR duplicates marked using Picard. Base Q score recalibration was performed using a machine learning technique to increase the accuracy of individual base quality scores. Variants were called using Mutect2^3^. Somatic mutations were defined using matched constitutional DNA where available. For cases with no matched DNA, a pool of 33 constitutional DNAs from paediatric B-NHL patients with no other underlying conditions was used as the reference. Somatic variants were identified and annotated using Ensembl Variant Effect Predictor (VEP). Mutations were analysed against the Catalogue of Somatic Mutations in Cancer (COSMIC), the Single Nucleotide Polymorphism (dbSNP) database and the Exome Aggregation Consortium (ExAC) database to remove germline variants. WES data were visualised in Integrative Genomics Viewer (IGV)^4^ and integrated with copy number data in Nexus Copy Number 10.0 (BioDiscovery).

All array and sequence data generated in the study has been deposited at the European Genome-phenome Archive (EGA), which is hosted by the EBI and the CRG, under accession number EGAS00001005617.

*Sanger Sequencing*

PCR products were amplified using the SureCycler 8800 (Agilent, UK), then purified using the QIAquick PCR Purification Kit (Qiagen) and sequenced by Eurofins Genomics. ABI format files were analyzed using GeneScreen and visualized using FinchTV software (Geospiza)^5^. Heterozygous mutation calls required a >0.2 peak height ratio and all mutated bases had a quality score >30. Each ABI chromatogram was manually checked to ensure the absence of background noise, ‘dye blobs’, low signal or discordant mutation calls between forward and reverse reads.

*Fluorescence in situ Hybridisation (FISH)*

FISH was performed by the Newcastle Genetics Laboratory, Newcastle upon Tyne Hospitals NHS Foundation Trust. 3-4μm FFPE tissue sections or imprint slides made from FF tumour tissue were used. FFPE sections were pre-treated using the Aquarius Tissue Pre-treatment Kit (Cytocell). Slides were hybridised with a P53 Deletion Probe (Cytocell), containing a 161kb probe against *TP53* and flanking regions and a chromosome 17 centromere probe. Denaturation, hybridisation and washing were performed according to the manufacturer’s instructions with minor modifications. Analysis was performed on a Leica DM5500B fluorescent microscope by experienced cytogeneticists.

*p53 Immunohistochemistry*

p53 protein expression was evaluated by immunohistochemistry. Tissue sections were stained for p53 using the Ventana Confirm anti-p53 mouse monoclonal antibody DO-7. Staining was performed on a Ventana Benchmark automated stainer according to the manufacturer’s instructions (Roche).

*Statistical Analysis*

Survival analysis was performed using two metrics: progression free survival (PFS) defined as the time from diagnosis to time of disease progression (relapse or refractory disease) or death and overall survival (OS), defined as the time from diagnosis to death, with censoring at the date of last contact. Clinical risk groups were defined as low (Stage I, Stage II tumour completely resected), intermediate (stage II (incompletely resected), stage III AND LDH <2 ULN) or high-risk (stage III AND LDH >2 ULN or CNS positive, stage IV (any LDH)). Kaplan Meier analysis of covariates for visualisation was performed by the log-rank method using the ‘survminer’ package in R. Univariate and multivariate Cox-regression modelling followed by the Wald test were applied to determine the statistical associations between clinical and genomic factors and survival using the ‘survival’ package in R. *TP53* abnormality covariates were compared against known clinical risk factors. Comparison of the incidence of covariate events between patient subgroups was performed using Fisher’s Exact test in R. Comparison of the proportion of the genome altered and the number of copy number segments altered between subgroups was performed using Student’s t-test in R.

**References**

1. McKenna A, Hanna M, Banks E, Sivachenko A, Cibulskis K, Kernytsky A*, et al.* The Genome Analysis Toolkit: a MapReduce framework for analyzing next-generation DNA sequencing data. *Genome research* 2010 Sep; **20**(9)**:** 1297-1303.

2. Li H, Durbin R. Fast and accurate short read alignment with Burrows-Wheeler transform. *Bioinformatics* 2009 Jul 15; **25**(14)**:** 1754-1760.

3. Cibulskis K, Lawrence MS, Carter SL, Sivachenko A, Jaffe D, Sougnez C*, et al.* Sensitive detection of somatic point mutations in impure and heterogeneous cancer samples. *Nat Biotechnol* 2013 Mar; **31**(3)**:** 213-219.

4. Robinson JT, Thorvaldsdottir H, Winckler W, Guttman M, Lander ES, Getz G*, et al.* Integrative genomics viewer. *Nat Biotechnol* 2011 Jan; **29**(1)**:** 24-26.

5. Carr IM, Camm N, Taylor GR, Charlton R, Ellard S, Sheridan EG*, et al.* GeneScreen: a program for high-throughput mutation detection in DNA sequence electropherograms. *J Med Genet* 2011 Feb; **48**(2)**:** 123-130.
