# Supplementary material for: Genomic abnormalities of TP53 define distinct risk groups of paediatric B-cell non-Hodgkin lymphoma
Source: Leukemia. 2021 Oct 21;36(3):781–9. doi: 10.1038/s41375-021-01444-6 (PMC8885412; doi:10.1038/s41375-021-01444-6)
Supplement: Supplementary file 2 — Supplemental Tables [file 41375_2021_1444_MOESM2_ESM.docx]

**Supplemental Tables**

**Supplemental Tables**

**Supplemental Table 1: *TP53* primers.**

| **Target Exon** | **Forward sequence** | **Reverse sequence** |
| --- | --- | --- |
| Exon 5 | CTCTGTCTCCTTCCTCTTCC | GCAATCAGTGAGGAATCAGAGG |
| Exon 6 | GCCTCTGATTCCTCACTGAT | GGAGGGCCACTGACAACCA |
| Exon 7 | AGGCGCACTGGCCTCATCTT | CAGGGGTCAGAGGCAAGCAGA |
| Exon 8 | GAGCCTGGTTTTTTAAATGG | TTTGGCTGGGGAGAGGAGCT |

**Supplemental Table 2: *TP53* abnormalities identified in paediatric B-NHL patient samples at diagnosis.**

| **ID** | **Diagnosis** | **17p copy number abnormality** | **Size of 17p alteration (Mb)** | **% of 17p altered** | ***TP53* Deletion FISH Result** | **Mutation (protein)** | **Mutation (nucleotide)** | **VAF (%)** | **Activity*** | **Functional classification**** | **Mutation type** | ***TP53* status** |
| --- | --- | --- | --- | --- | --- | --- | --- | --- | --- | --- | --- | --- |
| BL1 | BL | no abnormality |  |  | Concordant | no mutation |  |  |  |  | no mutation | no abnormality |
| BL2 | BL | 17p deletion | 22.2 | 99.6% | Concordant | p.G266E | c.797G>A | 85 | inactive | non-functional | hemizygous | biallelic |
| BL3 | BL | no abnormality |  |  | Concordant | p.R249T | c.746G>C | 21 | inactive | non-functional | heterozygous | monoallelic |
| BL4 | BL | 17p deletion | 20.5 | 91.9% | ND | no mutation |  |  |  |  | no mutation | monoallelic |
| BL5 | BL | no abnormality |  |  | ND | no mutation |  |  |  |  | no mutation | no abnormality |
| BL6 | BL | no abnormality |  |  | ND | p.D208G | c.623A>G | 39 | fully active | functional | heterozygous | monoallelic |
| BL7 | BL | no abnormality |  |  | ND | no mutation |  |  |  |  | no mutation | no abnormality |
| BL8 | BL | no abnormality |  |  | ND | p.S241P | c.721T>C | 38 | inactive | non-functional | heterozygous | monoallelic |
| BL9 | BL | no abnormality |  |  | Concordant | no mutation |  |  |  |  | no mutation | no abnormality |
| BL10 | BL | no abnormality |  |  | Concordant | no mutation |  |  |  |  | no mutation | no abnormality |
| BL11 | BL | no abnormality |  |  | Concordant | no mutation |  |  |  |  | no mutation | no abnormality |
| BL12 | BL | no abnormality |  |  | ND | p.L289fs*56 | c.866_867del | 89 | activity of truncated p53 is assumed to be nil | frameshift | homozygous | biallelic |
| BL13 | BL | no abnormality |  |  | Concordant | p.R248Q | c.743G>A | 44 | inactive | non-functional | heterozygous | monoallelic |
| BL14 | BL | no abnormality |  |  | Concordant | no mutation |  |  |  |  | no mutation | no abnormality |
| BL15 | BL | no abnormality |  |  | Concordant | no mutation |  |  |  |  | no mutation | no abnormality |
| BL16 | BL | no abnormality |  |  | Concordant | p.E258D | c.774A>C | 48 | inactive | non-functional | heterozygous | monoallelic |
| BL17 | BL | no abnormality |  |  | Concordant | p.R158H, p.R175H | c.473G>A, c.524G>A | 54, 36 | inactive, inactive | non-functional, non-functional | compound heterozygous | biallelic |
| BL18 | BL | no abnormality |  |  | Concordant | no mutation |  |  |  |  | no mutation | no abnormality |
| BL19 | BL | 17p deletion | 19.7 | 88.3% | Concordant | p.V122fs*2 | c.362del | 40 | activity of truncated p53 is assumed to be nil | frameshift | hemizygous | biallelic |
| BL20 | BL | no abnormality |  |  | Concordant | p.R175H, p.G245S | c.524G>A, c.733G>A | 49, 36 | inactive, inactive | non-functional, non-functional | compound heterozygous | biallelic |
| BL21 | BL | no abnormality |  |  | Concordant | p.V173M | c.517G>A | 36 | inactive | non-functional | heterozygous | monoallelic |
| BL22 | BL | no abnormality |  |  | Concordant | no mutation |  |  |  |  | no mutation | no abnormality |
| BL23 | BL | no abnormality |  |  | Concordant | p.G245S | c.733G>A | 39 | inactive | non-functional | heterozygous | monoallelic |
| BL24 | BL | no abnormality |  |  | Concordant | p.R158H, p.P278S | c.473G>A, c.832C>T | 53, 39 | inactive, inactive | non-functional, non-functional | compound heterozygous | biallelic |
| BL25 | BL | no abnormality |  |  | Concordant | no mutation |  |  |  |  | no mutation | no abnormality |
| BL26 | BL | no abnormality |  |  | Concordant | p.R248Q, p.R273C | c.743G>A, c.817C>T | 44, 20 | inactive, inactive | non-functional, non-functional | compound heterozygous | biallelic |
| BL27 | BL | no abnormality |  |  | Concordant | no mutation |  |  |  |  | no mutation | no abnormality |
| BL28 | BL | no abnormality |  |  | Concordant | p.Y205F | c.614A>T | 39 | inactive | non-functional | heterozygous | monoallelic |
| BL29 | BL | no abnormality |  |  | Concordant | no mutation |  |  |  |  | no mutation | no abnormality |
| BL30 | BL | no abnormality |  |  | Concordant | no mutation |  |  |  |  | no mutation | no abnormality |
| BL31 | BL | 17p CNN-LOH | 22.2 | 99.6% | Concordant | p.R248Q | c.743G>A | 40 | inactive | non-functional | homozygous | biallelic |
| BL32 | BL | no abnormality |  |  | Concordant | p.G266E | c.797G>A | 39 | inactive | non-functional | heterozygous | monoallelic |
| BL33 | BL | no abnormality |  |  | Concordant | no mutation |  |  |  |  | no mutation | no abnormality |
| BL34 | BL | no abnormality |  |  | Concordant | no mutation |  |  |  |  | no mutation | no abnormality |
| BL35 | BL | 17p deletion | 20.6 | 92.4% | ND | p.R175H | c.524G>A | 31 | inactive | non-functional | hemizygous | biallelic |
| BL36 | BL | no abnormality |  |  | Concordant | no mutation |  |  |  |  | no mutation | no abnormality |
| BL37 | BL | 17p deletion | 17.9 | 80.3% | Concordant | p.V216G | c.647T>G | 92 | inactive | non-functional | hemizygous | biallelic |
| BL38 | BL | 17p deletion | 20.6 | 92.4% | Concordant | p.D281G | c.842A>G | 24 | inactive | non-functional | hemizygous | biallelic |
| BL39 | BL | no abnormality |  |  | ND | no mutation |  |  |  |  | no mutation | no abnormality |
| BL40 | BL | no abnormality |  |  | Concordant | no mutation |  |  |  |  | no mutation | no abnormality |
| BL41 | BL | no abnormality |  |  | Concordant | p.R282W | c.844C>T | 18 | inactive | non-functional | heterozygous | monoallelic |
| BL42 | BL | no abnormality |  |  | Concordant | p.Y126H, p.G245S | c.376T>C, c.733G>A | 47, 42 | inactive, inactive | non-functional, non-functional | compound heterozygous | biallelic |
| BL43 | BL | 17p deletion | 22.2 | 99.6% | Concordant | p.R273H | c.818G>A | 59 | inactive | non-functional | hemizygous | biallelic |
| BL44 | BL | no abnormality |  |  | ND | p.R248Q | c.743G>A | 41 | inactive | non-functional | heterozygous | monoallelic |
| BL45 | BL | no abnormality |  |  | Concordant | p.C135R | c.403T>C | 38 | inactive | non-functional | heterozygous | monoallelic |
| BL46 | BL | no abnormality |  |  | Concordant | p.R175H | c.524G>A | 50 | inactive | non-functional | heterozygous | monoallelic |
| BL47 | BL | 17p CNN-LOH | 14 | 62.8% | Concordant | p.T155N | c.464C>A | 84 | inactive | non-functional | homozygous | biallelic |
| BL48 | BL | 17p CNN-LOH | 21.8 | 97.8% | ND | p.G245S | c.733G>A | 88 | inactive | non-functional | homozygous | biallelic |
| BL49 | BL | no abnormality |  |  | ND | no mutation |  |  |  |  | no mutation | no abnormality |
| BL50 | BL | no abnormality |  |  | ND | no mutation |  |  |  |  | no mutation | no abnormality |
| BL51 | BL | 17p CNN-LOH | 17.2 | 77.1% | Concordant | p.R175H | c.524G>A | 91 | inactive | non-functional | homozygous | biallelic |
| BL52 | BL | no abnormality |  |  | ND | p.R333P | c.998G>C | 36 | partial activity | partially functional | heterozygous | monoallelic |
| BL53 | BL | no abnormality |  |  | Concordant | no mutation |  |  |  |  | no mutation | no abnormality |
| BL54 | BL | 17p deletion | 22.2 | 99.6% | Concordant | p.R196P | c.587G>C | 88 | inactive | non-functional | hemizygous | biallelic |
| BL55 | BL | 17p CNN-LOH | 12.9 | 57.8% | Concordant | no mutation |  |  |  |  | no mutation | monoallelic |
| BL56 | BL | no abnormality |  |  | Concordant | p.R248Q, p.T256K | c.743G>A, c.767C>A | 41, 40 | inactive, inactive | partially functional, non-functional | compound heterozygous | biallelic |
| BL57 | BL | no abnormality |  |  | ND | p.R273C | c.817C>T | 45 | inactive | non-functional | heterozygous | monoallelic |
| BL58 | BL | no abnormality |  |  | Concordant | no mutation |  |  |  |  | no mutation | no abnormality |
| BL59 | BL | 17p deletion | 18.5 | 83.0% | Concordant | no mutation |  |  |  |  | no mutation | monoallelic |
| BL60 | BL | no abnormality |  |  | Concordant | no mutation |  |  |  |  | no mutation | no abnormality |
| BL61 | BL | 17p deletion | 19.4 | 87.0% | Concordant | p.R273C | c.817C>T | 86 | inactive | non-functional | hemizygous | biallelic |
| BL62 | BL | no abnormality |  |  | Concordant | p.P151A | c.451C>G | 20 | inactive | non-functional | heterozygous | monoallelic |
| BL63 | BL | 17p CNN-LOH | 22.2 | 99.6% | ND | p.R175H | c.524G>A | 92 | inactive | non-functional | homozygous | biallelic |
| BL64 | BL | 17p CNN-LOH | 16.4 | 73.5% | Concordant | p.H179R | c.536A>G | 92 | inactive | non-functional | homozygous | biallelic |
| DLBCL1 | DLBCL | no abnormality |  |  | Concordant | no mutation |  |  |  |  | no mutation | no abnormality |
| DLBCL2 | DLBCL | no abnormality |  |  | Concordant | no mutation |  |  |  |  | no mutation | no abnormality |
| DLBCL3 | DLBCL | 17p deletion | 22.2 | 99.6% | Concordant | no mutation |  |  |  |  | no mutation | monoallelic |
| DLBCL4 | DLBCL | no abnormality |  |  | Concordant | p.R248W | c.742C>T | 49 | inactive | non-functional | heterozygous | monoallelic |
| DLBCL5 | DLBCL | no abnormality |  |  | Concordant | no mutation |  |  |  |  | no mutation | no abnormality |
| DLBCL6 | DLBCL | no abnormality |  |  | Concordant | no mutation |  |  |  |  | no mutation | no abnormality |
| DLBCL7 | DLBCL | 17p CNN-LOH | 22.3 | 100.0% | ND | no mutation |  |  |  |  | no mutation | monoallelic |
| DLBCL8 | DLBCL | no abnormality |  |  | Concordant | no mutation |  |  |  |  | no mutation | no abnormality |
| DLBCL9# | DLBCL | 17p deletion | 21.5 | 96.4% | Concordant | p.R248Q | c.743G>A | 56 | inactive | non-functional | hemizygous | biallelic |
| DLBCL10 | DLBCL | 17p deletion | 19 | 85.2% | Concordant | no mutation |  |  |  |  | no mutation | monoallelic |
| DLBCL11 | DLBCL | no abnormality |  |  | Concordant | no mutation |  |  |  |  | no mutation | no abnormality |
| DLBCL12 | DLBCL | no abnormality |  |  | Concordant | no mutation |  |  |  |  | no mutation | no abnormality |
| DLBCL13 | DLBCL | no abnormality |  |  | Concordant | no mutation |  |  |  |  | no mutation | no abnormality |
| DLBCL14 | DLBCL | no abnormality |  |  | Concordant | no mutation |  |  |  |  | no mutation | no abnormality |
| DLBCL15 | DLBCL | no abnormality |  |  | Concordant | no mutation |  |  |  |  | no mutation | no abnormality |
| DLBCL16 | DLBCL | 17p deletion | 22.2 | 99.6% | ND | p.R213Q | c.638G>A | 71 | inactive | non-functional | hemizygous | biallelic |
| DLBCL17 | DLBCL | no abnormality |  |  | Concordant | no mutation |  |  |  |  | no mutation | no abnormality |
| DLBCL18 | DLBCL | no abnormality |  |  | ND | no mutation |  |  |  |  | no mutation | no abnormality |
| DLBCL19 | DLBCL | no abnormality |  |  | Concordant | p.G187S | c.559G>A | 39 | fully active | functional | heterozygous | monoallelic |
| BLL-11q1 | BLL-11q | no abnormality |  |  | Concordant | no mutation |  |  |  |  | no mutation | no abnormality |
| BLL-11q2 | BLL-11q | no abnormality |  |  | Concordant | no mutation |  |  |  |  | no mutation | no abnormality |
| BLL-11q3 | BLL-11q | no abnormality |  |  | Concordant | no mutation |  |  |  |  | no mutation | no abnormality |
| BLL-11q4 | BLL-11q | no abnormality |  |  | Concordant | no mutation |  |  |  |  | no mutation | no abnormality |
| BLL-11q5 | BLL-11q | no abnormality |  |  | Concordant | p.C176F | c.527G>T | 40 | inactive | partially functional | heterozygous | monoallelic |
| B-NHL1 | B-NHL, NOS | no abnormality |  |  | Concordant | p.E286K | c.856G>A | 41 | inactive | non-functional | heterozygous | monoallelic |
| B-NHL2 | B-NHL, NOS | no abnormality |  |  | ND | no mutation |  |  |  |  | no mutation | no abnormality |
| B-NHL3 | B-NHL, NOS | no abnormality |  |  | Concordant | no mutation |  |  |  |  | no mutation | no abnormality |
| B-NHL4 | B-NHL, NOS | no abnormality |  |  | Concordant | p.S241Y, p.G245S | c.722C>A, c.733G>A | 32, 22 | inactive, inactive | non-functional, non-functional | compound heterozygous | biallelic |
| B-NHL5 | B-NHL, NOS | no abnormality |  |  | Concordant | p.Y126S | c.377A>C | 96 | inactive | non-functional | homozygous | biallelic |
| B-NHL6 | B-NHL, NOS | no abnormality |  |  | Concordant | no mutation |  |  |  |  | no mutation | no abnormality |
| B-NHL7 | B-NHL, NOS | 17p deletion | 22.2 | 99.6% | Concordant | p.L257P, p.R282W | c.770T>C, c.844C>T | 65, 30 | inactive, inactive | non-functional, non-functional | hemizygous | biallelic |

BL = Burkitt lymphoma; DLBCL = diffuse large B-cell lymphoma; BLL-11q = Burkitt-like lymphoma with 11q aberrations; B-NHL, NOS = B-cell non-Hodgkin lymphoma not otherwise specified; ND = not done; VAF, variant allele frequency.

* Data obtained from the UMD TP53 database (http://p53.fr/the-database)^6^.

** Functional classification based on transcriptional activity (TA) obtained from IARC *TP53* Database (http://p53.iarc.fr/TP53GeneVariations.aspx)^7^

# Li-Fraumeni syndrome patient with germline *TP53* mutation.

**Supplemental Table 3: Comparison of *TP53* genomic status and p53 protein expression detected by immunohistochemical (IHC) staining**

| **ID** | ***TP53* status** | ***TP53* Mutation(s)** | **Tumour cell p53 expression by immunohistochemistry** |
| --- | --- | --- | --- |
| BL1 | WT | no mutation | 80% positive; variable intensity (many strong) |
| BL15 | WT | no mutation | 70% positive; variable intensity (many strong) |
| DLBCL5 | WT | no mutation | 30% positive; variable intensity (some strong) |
| BL14 | WT | no mutation | 50% positive; weak |
| BL33 | WT | no mutation | 40% positive; weak |
| BL36 | WT | no mutation | 30% positive; weak |
| BL34 | WT | no mutation | 20% positive; weak |
| BL27 | WT | no mutation | 20% positive; weak |
| DLBCL6 | WT | no mutation | <5% positive; weak |
| BL29 | WT | no mutation | <5% positive; weak |
| BL17 | Biallelic | p.R158H, p.R175H | 90% positive; strong |
| BL54 | Biallelic | p.R196P | 90% positive; strong |
| DLBCL9 | Biallelic | p.R248Q | 80% positive; strong |
| BLL-11q5 | Monoallelic | p.C176F | 80% positive; moderate / strong |
| BL28 | Monoallelic | p.Y205F | 80% positive; moderate |
| BL39 reassessment | Biallelic | p.R248Q | 50% positive; strong |
| DLBCL4 | Monoallelic | p.E258D | 50% positive; variable intensity (some strong) |
| BL16 | Monoallelic | p.R248W | 50% positive; variable intensity (some strong) |
| BL31 | Biallelic | p.R248Q | 20% positive; strong (patchy) |
| BL13 | Monoallelic | p.R248Q | <5% positive; variable intensity (some strong) |

WT = wild-type; IHC = immunohistochemistry

**Supplemental Table 4: Genomic complexity in paediatric B-NHL with or without *TP53* abnormalities at diagnosis.**

| ***TP53* status** | **Mean % genome altered** | ***p* value** | **Mean number of CNAs** | ***p* value** | **Mean number of chromosomes with complex CNA patterns** | ***p* value** |
| --- | --- | --- | --- | --- | --- | --- |
| ***TP53* abnormal** | 6.44 | 0.378 | 48.13 | 0.085 | 1.04 | 0.004 |
| ***Biallelic*** | 6.04 | 0.442 | 39.62 | 0.233 | 1.58 | 0.007 |
| ***Monoallelic*** | 6.85 | 0.530 | 56.65 | 0.162 | 0.50 | 0.145 |
| ***TP53* normal** | 4.57 | - | 29.49 | - | 0.28 | - |

*P* values compare *TP53* altered groups with *TP53* normal group (Student’s t-test). CNA = copy number abnormalities.

**Supplemental Table 5: *IG-MYC* rearrangements, *TP53* status and chromosomes with complex copy number profiles identified in paediatric B-NHL patient samples at diagnosis.**

| **ID** | **Diagnosis** | ***IG-MYC* status** | ***TP53 s*tatus** | **Chromosome complexity** | **Chromosomes with complex copy number patterns** |
| --- | --- | --- | --- | --- | --- |
| BL1 | BL | *IG-MYC* | no abnormality | yes | 1q, 2p, 13q^plex^ |
| BL2 | BL | *IG-MYC* | biallelic | no |  |
| BL3 | BL | *IG-MYC* | monoallelic | no |  |
| BL4 | BL | *IG-MYC* | monoallelic | yes | 13q |
| BL5 | BL | fail/NA | no abnormality | no |  |
| BL6 | BL | *IG-MYC* | monoallelic | no |  |
| BL7 | BL | fail/NA | no abnormality | no |  |
| BL8 | BL | *IG-MYC* | monoallelic | no |  |
| BL9 | BL | *IG-MYC* | no abnormality | no |  |
| BL10 | BL | *IG-MYC* | no abnormality | no |  |
| BL11 | BL | *IG-MYC* | no abnormality | no |  |
| BL12 | BL | *IG-MYC* | biallelic | yes | 1q, 2p, 7q |
| BL13 | BL | *IG-MYC* | monoallelic | yes | 1q |
| BL14 | BL | *IG-MYC* | no abnormality | no |  |
| BL15 | BL | *IG-MYC* | no abnormality | no |  |
| BL16 | BL | *IG-MYC* | monoallelic | no |  |
| BL17 | BL | *IG-MYC* | biallelic | no |  |
| BL18 | BL | *IG-MYC* | no abnormality | no |  |
| BL19 | BL | *IG-MYC* | biallelic | yes | 13q^plex^ |
| BL20 | BL | *IG-MYC* | biallelic | yes | 13q^plex^ |
| BL21 | BL | *IG-MYC* | monoallelic | yes | 3q, 10q |
| BL22 | BL | *IG-MYC* | no abnormality | no |  |
| BL23 | BL | *IG-MYC* | monoallelic | no |  |
| BL24 | BL | *IG-MYC* | biallelic | yes | 13q |
| BL25 | BL | no *MYC* translocation | no abnormality | no |  |
| BL26 | BL | *IG-MYC* | biallelic | yes | 12q |
| BL27 | BL | *IG-MYC* | no abnormality | no |  |
| BL28 | BL | *IG-MYC* | monoallelic | yes | 7q |
| BL29 | BL | *IG-MYC* | no abnormality | no |  |
| BL30 | BL | *IG-MYC* | no abnormality | no |  |
| BL31 | BL | *IG-MYC* | biallelic | yes | 1q, 11q |
| BL32 | BL | *IG-MYC* | monoallelic | yes | 1q |
| BL33 | BL | *IG-MYC* | no abnormality | no |  |
| BL34 | BL | *IG-MYC* | no abnormality | no |  |
| BL35 | BL | *IG-MYC* | biallelic | yes | 13q^plex^, 17p |
| BL36 | BL | no *MYC* translocation | no abnormality | no |  |
| BL37 | BL | *IG-MYC* | biallelic | no |  |
| BL38 | BL | *IG-MYC* | biallelic | no |  |
| BL39 | BL | *IG-MYC* | no abnormality | no |  |
| BL40 | BL | *IG-MYC* | no abnormality | no |  |
| BL41 | BL | *IG-MYC* | monoallelic | yes | 1p |
| BL42 | BL | *IG-MYC* | biallelic | no |  |
| BL43 | BL | *IG-MYC* | biallelic | yes | 13q^plex^ |
| BL44 | BL | *IG-MYC* | monoallelic | yes | 1q |
| BL45 | BL | *IG-MYC* | monoallelic | yes | 6q |
| BL46 | BL | *IG-MYC* | monoallelic | no |  |
| BL47 | BL | *IG-MYC* | biallelic | yes | 7q, 11q, 13q |
| BL48 | BL | *IG-MYC* | biallelic | yes | 8q, 13q^plex^ |
| BL49 | BL | *IG-MYC* | no abnormality | no |  |
| BL50 | BL | *IG-MYC* | no abnormality | no |  |
| BL51 | BL | *IG-MYC* | biallelic | yes | 11q, 13q^plex^ |
| BL52 | BL | *IG-MYC* | monoallelic | no |  |
| BL53 | BL | *IG-MYC* | no abnormality | no |  |
| BL54 | BL | *IG-MYC* | biallelic | yes | 11q, 13q^plex^ |
| BL55 | BL | *IG-MYC* | monoallelic | no |  |
| BL56 | BL | *IG-MYC* | biallelic | no |  |
| BL57 | BL | *IG-MYC* | monoallelic | yes | 1q |
| BL58 | BL | *IG-MYC* | no abnormality | no |  |
| BL59 | BL | *IG-MYC* | monoallelic | yes | 3q |
| BL60 | BL | *IG-MYC* | no abnormality | no |  |
| BL61 | BL | *IG-MYC* | biallelic | yes | 13q^plex^ |
| BL62 | BL | *IG-MYC* | monoallelic | no |  |
| BL63 | BL | *IG-MYC* | biallelic | yes | 13q^plex^ |
| BL64 | BL | *IG-MYC* | biallelic | no |  |
| DLBCL1 | DLBCL | no *MYC* translocation | no abnormality | no |  |
| DLBCL2 | DLBCL | no *MYC* translocation | no abnormality | no |  |
| DLBCL3 | DLBCL | no *MYC* translocation | monoallelic | no |  |
| DLBCL4 | DLBCL | *IG-MYC* | monoallelic | no |  |
| DLBCL5 | DLBCL | no *MYC* translocation | no abnormality | no |  |
| DLBCL6 | DLBCL | *IG-MYC* | no abnormality | no |  |
| DLBCL7 | DLBCL | no *MYC* translocation | monoallelic | yes | 6p |
| DLBCL8 | DLBCL | no *MYC* translocation | no abnormality | yes | 13q |
| DLBCL9^#^ | DLBCL | no *MYC* translocation | biallelic | yes | 4q, 8q, 11p, 11q, 12, 14p, 14q, 15q, 17p, 17q |
| DLBCL10 | DLBCL | no *MYC* translocation | monoallelic | no |  |
| DLBCL11 | DLBCL | no *MYC* translocation | no abnormality | no |  |
| DLBCL12 | DLBCL | no *MYC* translocation | no abnormality | yes | 6q, 9q |
| DLBCL13 | DLBCL | fail/NA | no abnormality | no |  |
| DLBCL14 | DLBCL | fail/NA | no abnormality | no |  |
| DLBCL15 | DLBCL | no *MYC* translocation | no abnormality | no |  |
| DLBCL16 | DLBCL | *IG-MYC* | biallelic | no |  |
| DLBCL17 | DLBCL | no *MYC* translocation | no abnormality | no |  |
| DLBCL18 | DLBCL | no *MYC* translocation | no abnormality | yes | 1p |
| DLBCL19 | DLBCL | *IG-MYC* | monoallelic | no |  |
| BLL-11q1 | BLL-11q | no *MYC* translocation | no abnormality | BLL-11q | 11q |
| BLL-11q2 | BLL-11q | no *MYC* translocation | no abnormality | BLL-11q | 11q |
| BLL-11q3 | BLL-11q | no *MYC* translocation | no abnormality | BLL-11q | 11q |
| BLL-11q4 | BLL-11q | no *MYC* translocation | no abnormality | BLL-11q | 11q |
| BLL-11q5 | BLL-11q | no *MYC* translocation | monoallelic | yes | 11q |
| B-NHL1 | B-NHL, NOS | *IG-MYC* | monoallelic | no |  |
| B-NHL2 | B-NHL, NOS | no *MYC* translocation | no abnormality | no |  |
| B-NHL3 | B-NHL, NOS | fail/NA | no abnormality | yes | 13q |
| B-NHL4 | B-NHL, NOS | *IG-MYC* | biallelic | yes | 1q |
| B-NHL5 | B-NHL, NOS | *IG-MYC* | biallelic | yes | 1q, 3q, 18q |
| B-NHL6 | B-NHL, NOS | no *MYC* translocation | no abnormality | no |  |
| B-NHL7 | B-NHL, NOS | *IG-MYC* | biallelic | yes | 1q, 2p, 10q |

BL = Burkitt lymphoma; DLBCL = diffuse large B-cell lymphoma; BLL-11q = Burkitt-like lymphoma with 11q aberration; B-NHL, NOS = B-cell non-Hodgkin lymphoma, not otherwise specified; NA = not available.

# Li-Fraumeni syndrome patient with germline *TP53* mutation.

**Supplemental Table 6: Clinical and cytogenetic characteristics of FAB/LMB96-treated paediatric B-NHL patients with and without *TP53* abnormalities.**

|  |  | **No *TP53* abnormality** |  | ***TP53* abnormal** | | | | | | | |
| --- | --- | --- | --- | --- | --- | --- | --- | --- | --- | --- | --- |
|  |  |  |  | **Any *TP53* abnormality** | |  | **Monoallelic abnormality** | |  | **Biallelic abnormality** | |
| **Total Cases** |  | **39** |  | **50** | ***p* value** |  | **25** | ***p* value** |  | **25** | ***p* value** |
| Diagnosis | BL | 20 |  | 40 | 0.006 |  | 19 | 0.067 |  | 21 | 0.009 |
|  | DLBCL | 12 |  | 6 | 0.036 |  | 4 | 0.243 |  | 2 | 0.036 |
|  | BLL-11q | 4 |  | 1 | 0.164 |  | 1 | 0.640 |  | 0 | 0.149 |
|  | B-NHL, NOS | 3 |  | 3 | 1 |  | 1 | 1 |  | 2 | 1.000 |
| Age |  | 8 (2-17) |  | 10 (0-16) |  |  | 10 (0-16) |  |  | 10 (1-15) |  |
| Sex | Male | 29 |  | 39 | 0.614 |  | 19 | 1 |  | 20 | 0.538 |
|  | Female | 10 |  | 10 |  |  | 6 |  |  | 4 |  |
|  | Not available | 0 |  | 1 |  |  | 0 |  |  | 1 |  |
| Tumour Stage | Stage I or II | 14 |  | 10 | 0.148 |  | 7 | 0.592 |  | 3 | 0.044 |
|  | Stage III or IV | 25 |  | 40 |  |  | 18 |  |  | 22 |  |
|  | Not available | 0 |  | 0 |  |  | 0 |  |  | 0 |  |
| BM Involvement | Y | 5 |  | 11 | 0.279 |  | 5 | 0.485 |  | 6 | 0.315 |
|  | N | 34 |  | 38 |  |  | 19 |  |  | 19 |  |
|  | Not available | 0 |  | 1 |  |  | 1 |  |  | 0 |  |
| CNS Involvement | Y | 2 |  | 3 | 1 |  | 2 | 0.632 |  | 1 | 1 |
|  | N | 37 |  | 46 |  |  | 22 |  |  | 24 |  |
|  | Not available | 0 |  | 1 |  |  | 1 |  |  | 0 |  |
| LDH > 2x ULN | Y | 13 |  | 25 | 0.130 |  | 10 | 0.569 |  | 15 | 0.005 |
|  | N | 21 |  | 17 |  |  | 10 |  |  | 7 |  |
|  | Not available | 5 |  | 8 |  |  | 5 |  |  | 3 |  |
| *MYC* Translocation | Y | 17 |  | 46 | <0.001 |  | 22 | 0.003 |  | 24 | <0.001 |
|  | N | 17 |  | 4 |  |  | 3 |  |  | 1 |  |
|  | Not available | 5 |  | 0 |  |  | 0 |  |  | 0 |  |
| Risk Group | High | 14 |  | 27 | 0.122 |  | 10 | 0.784 |  | 17 | 0.037 |
|  | Intermediate | 19 |  | 19 | 1.000 |  | 11 | 1 |  | 8 | 0.125 |
|  | Low | 3 |  | 1 | 0.312 |  | 1 | 1 |  | 0 | 0.262 |
|  | Not available | 3 |  | 3 |  |  | 3 |  |  | 0 |  |
| Treatment Group | Group A | 3 |  | 1 | 0.315 |  | 1 | 1 |  | 0 | 0.275 |
|  | Group B | 29 |  | 39 | 0.802 |  | 19 | 1 |  | 20 | 0.765 |
|  | Group C | 6 |  | 9 | 0.784 |  | 4 | 1 |  | 5 | 0.738 |
|  | Group unknown | 1 |  | 1 | 1 |  | 1 | 1 |  | 0 | 1 |
|  | Rituximab Added | 0 |  | 2 | 0.502 |  | 0 | 1 |  | 2 | 0.149 |
|  | No Rituximab | 38 |  | 46 |  |  | 23 |  |  | 23 |  |
|  | Rituximab unknown | 1 |  | 2 |  |  | 2 |  |  | 0 |  |

BL = Burkitt lymphoma; DLBCL = diffuse large B-cell lymphoma; BLL-11q = Burkitt-like lymphoma with 11q aberration; HGBL, NOS = high-grade B-cell lymphoma, not otherwise specified; B-NHL, NOS = B-cell non-Hodgkin lymphoma not otherwise specified; Y = Yes; N= No; "-" = no event, hazard ratio not reported; BM = bone marrow; CNS = central nervous system; CSF = cerebrospinal fluid; LDH = lactate dehydrogenase; ULN = upper limit of normal. *P* values compare the *TP53* abnormal groups with the *TP53* normal group in the header.

**Supplemental Table 7: Univariate and multivariate Cox Regression analysis of survival.**

| **Variable** | **Progression-free survival (PFS)** | | **Overall survival (OS)** | |
| --- | --- | --- | --- | --- |
|  | **Hazard ratio (95% CI)** | ***p* value** | **Hazard ratio (95% CI)** | ***p* value** |
| ***Univariate analysis*** | | | | |
| Age* | 0.9 (0.8-1.1) | 0.357 | 1.0 (0.9-1.2) | 0.980 |
| Sex (male vs female) | 0.5 (0.2-1.6) | 0.244 | 0.5 (0.1-1.7) | 0.281 |
| CNS involvement | 3.0 (0.7-13.1) | 0.153 | 4.4 (1.0-20.4) | 0.058 |
| BM involvement | 3.3 (1.2-9.3) | 0.023 | 6.2 (1.9-20.5) | 0.003 |
| LDH > 2x ULN | 5.5 (1.2-25.2) | 0.027 | 3.8 (0.8-18.1) | 0.099 |
| Disease stage (stage III-IV vs stage I-II) | 2.8 (1.3-6.2) | 0.009 | 4.3 (1.6-11.9) | 0.005 |
| *TP53* deletion | 1.4 (0.4-5.0) | 0.597 | 0.5 (0.1-4.0) | 0.518 |
| *TP53* CNN-LOH | 4.9 (1.6-15.6) | 0.007 | 4.7 (1.2-17.7) | 0.023 |
| *TP53* mutation | - | - | - | - |
| Any *TP53* abnormality | - | - | - | - |
| *TP53* monoallelic abnormality | - | - | - | - |
| *TP53* biallelic abnormality | - | - | - | - |
| ***Multivariate analysis - Any TP53 abnormality*** | | | |  |
| Any *TP53* abnormality | - | - | - | - |
| CNS involvement | 3.0 (0.2-38.9) | 0.405 | 5.1 (0.4-72.7) | 0.229 |
| BM involvement | 1.8 (0.2-13.9) | 0.566 | 3.6 (0.4-35.1) | 0.266 |
| LDH > 2x ULN | 4.2 (0.6-31.2) | 0.161 | 1.4 (0.2-12.5) | 0.770 |
| Disease stage (stage III-IV vs stage I-II) | 6.9 (0.1-3.9) | 0.673 | 9.2 (0.1-6.3) | 0.933 |

CI = confidence interval; * continuous variable; “-" = no event in one group, hazard ratio not reported; BM = bone marrow; CNS = central nervous system; LDH = lactate dehydrogenase; ULN = upper limit of normal.

**Supplemental Table 8: Univariate Cox regression analysis of survival according to 1q, 11q and 13q chromosomal complexity.**

| **Risk Factor** | **Progression-free survival (PFS)** | | **Overall survival (OS)** | |
| --- | --- | --- | --- | --- |
|  | **Hazard ratio (95% CI)** | ***p* value** | **Hazard ratio (95% CI)** | ***p* value** |
| **Chromosomal complexity** | | |  |  |
| 1q chromosomal complexity | 2.5 (0.7-8.8) | 0.159 | 2.2 (0.5-10.3) | 0.306 |
| 11q chromosomal complexity | 0.5 (0.1-4.0) | 0.534 | - | - |
| 13q chromosomal complexity | 1.3 (0.4-4.4) | 0.734 | 1.1 (0.2-5.0) | 0.923 |
| *MIR17HG* gain | 1.3 (0.4-3.8) | 0.622 | 1.5 (0.4-5.2) | 0.506 |

CI = confidence interval; "-" = no event in one group, hazard ratio not reported

**Supplemental Table 9: *TP53* abnormalities in samples taken at initial diagnosis and disease progression.**

| **ID** | **Sample** | **Diagnosis** | **Copy number abnormality** | **Size of 17p alteration (Mb)** | **% of 17p altered** | ***TP53* Deletion FISH Result** | **Mutation (protein)** | **Mutation (nucleotide)** | **VAF %** | **Mutation type** | ***TP53* status** | **Time to event (mo)** | **Time to last follow up (mo)** | **Status at last follow up** |
| --- | --- | --- | --- | --- | --- | --- | --- | --- | --- | --- | --- | --- | --- | --- |
| BL2 | diagnosis | BL | deletion | 22.2 | 99.6 | Concordant | G266E | c.797G>A | 85 | hemizygous | biallelic | 3.4 | 17.3 | Alive |
|  | progression | BL | deletion | 18.3 | 82.1 | Concordant | G266E | c.797G>A | 92 | hemizygous | biallelic |  |  |  |
| BL37 | diagnosis | BL | deletion | 17.9 | 80.3 | Concordant | V216G | c.647T>G | 92 | hemizygous | biallelic | 4.3 | 7.1 | Dead |
|  | progression | BL | deletion | 21.3 | 95.5 | Concordant | V216G | c.647T>G | 86 | hemizygous | biallelic |  |  |  |
| BL26 | diagnosis | BL | no | - | - | Concordant | R248Q, R273C | c.743G>A, c.817C>T | 44, 20 | heterozygous | biallelic | 2.8 | 5.3 | Dead |
|  | progression | BL | no | - | - | Concordant | R248Q, R273C | c.743G>A, c.817C>T | 49, 44 | heterozygous | biallelic |  |  |  |
| BL31 | diagnosis | BL | CNN-LOH | 22.3 | 100 | Concordant | R248Q | c.743G>A | 40 | homozygous | biallelic | 6.1 | 123.1 | Dead |
|  | progression | BL | CNN-LOH | 22.3 | 100 | Concordant | R248Q | c.743G>A | NA | homozygous | biallelic |  |  |  |
| BL39 | diagnosis | BL | no | - | - | NA | No mutation | - | - | - | no abnormality | 3.2 | 45.7 | Alive |
|  | reassessment | BL | deletion | 18.5 | 83.0 | Concordant | R248W | c.742C>T | 54 | hemizygous | biallelic |  |  |  |
| BL23 | diagnosis | BL | no | - | - | Concordant | G245S | c.733G>A | 39 | heterozygous | monoallelic | 5.8 | 11.1 | Alive |
|  | progression | BL | CNN-LOH | 11 | 49.3 | Concordant | G245S | c.733G>A | NA | homozygous | biallelic |  |  |  |
| DLBCL7 | diagnosis | DLBCL | CNN-LOH | 22.1 | 99.1 | Concordant | No mutation | - | - | - | monoallelic | 8.2 | 17.1 | Dead |
|  | progression | DLBCL | CNN-LOH | 22.3 | 100 | Concordant | No mutation | - | - | - | monoallelic |  |  |  |
| DLBCL19 | diagnosis | DLBCL | no | - | - | Concordant | G187S | c.559G>A | 39 | heterozygous | monoallelic | 7.7 | 36.0 | Alive |
|  | progression | DLBCL | no | - | - | NA | G187S | c.559G>A | 20 | heterozygous | monoallelic |  |  |  |

BL = Burkitt lymphoma; DLBCL = diffuse large B-cell lymphoma; VAF = variant allele frequency; NA = not available; mo= month.
