# Supplementary material for: Genomic abnormalities of TP53 define distinct risk groups of paediatric B-cell non-Hodgkin lymphoma
Source: Leukemia. 2021 Oct 21;36(3):781–9. doi: 10.1038/s41375-021-01444-6 (PMC8885412; doi:10.1038/s41375-021-01444-6)
Supplement: Supplementary file 3 — Supplemental Figure Legends [file 41375_2021_1444_MOESM3_ESM.docx]

**Supplemental Figures**

**Supplemental Figure 1: Complex chromosomal copy number abnormalities of 1q.** (A) Patterns of complex copy number abnormality of chromosome arm 1q in 10 cases. (B) Three examples of cases showing complex copy number patterns. Regions of gain are shaded in blue and deletions in red.

**Supplemental Figure 2: Complex chromosomal copy number abnormalities of 11q.** (A) Patterns of complex copy number abnormality of chromosome arm 11q in 10 cases. (B) Three examples of cases showing complex copy number patterns. Regions of gain are shaded in blue and deletions in red.

**Supplemental Figure 3: Complex chromosomal copy number abnormalities of 13q with recurrent gain/amplification of *MIR17HG*.** (A) Patterns of complex copy number abnormality of 13q in 10 BL cases with 13q^plex^ abnormalities and 5 cases with other 13q complex abnormalities. (B) Three examples of cases showing complex copy number patterns of 13q. Regions of gain are shaded in blue and deletions in red.

**Supplemental Figure 4: Prognostic significance of *TP53* mutations and copy number abnormalities in paediatric B-NHL** Kaplan-Meier plots showing progression free survival and overall survival for patients with or without *TP53* mutation (A-B), with or without *TP53* CNN-LOH (C-D), and with or without *TP53* deletion (E-F).

**Supplemental Figure 5: Prognostic significance of *TP53* abnormalities in paediatric BL** Kaplan-Meier plots showing progression free survival (A) and overall survival (B) for patients with or without *TP53* abnormalities.

**Supplemental Figure 6: Prognostic significance of *TP53* abnormalities in intermediate-risk paediatric B-NHL** Kaplan-Meier plots showing progression free survival (A) and overall survival (B) for patients with or without *TP53* abnormalities.
