# Supplementary figures and images for: Genomic abnormalities of TP53 define distinct risk groups of paediatric B-cell non-Hodgkin lymphoma
Source: Leukemia. 2021 Oct 21;36(3):781–9. doi: 10.1038/s41375-021-01444-6 (PMC8885412; doi:10.1038/s41375-021-01444-6)

A.

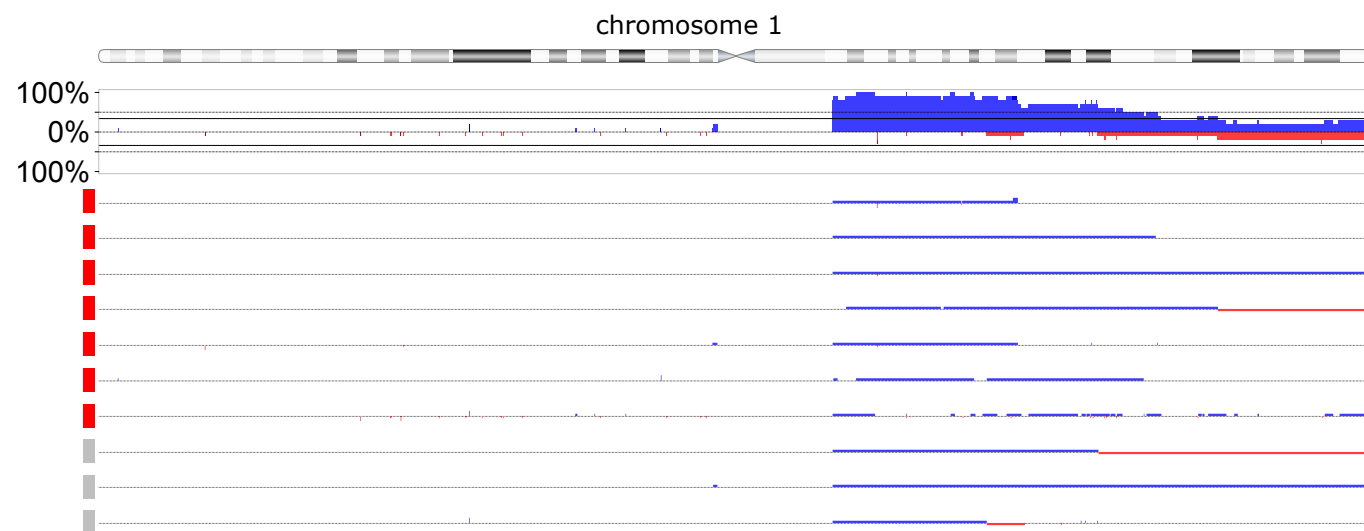

B.

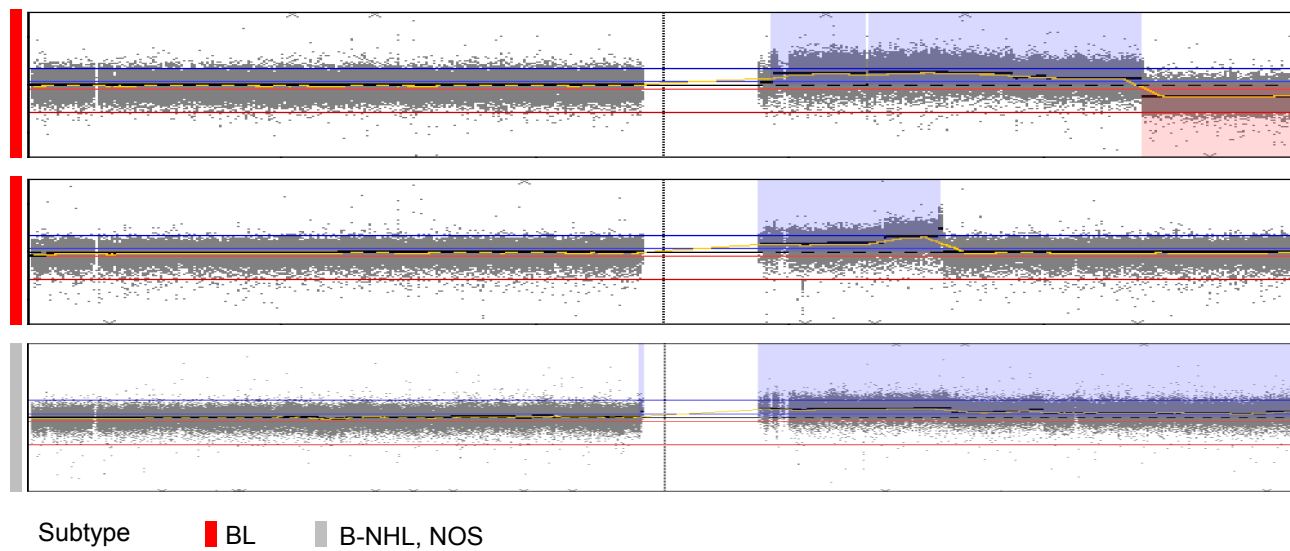

Supplement: Supplementary file 4 — Supplemental Figure 1 [file 41375_2021_1444_MOESM4_ESM.pdf]

A.

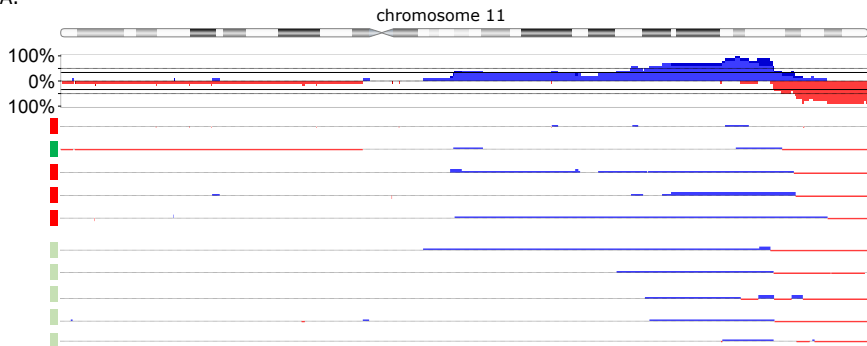

B.

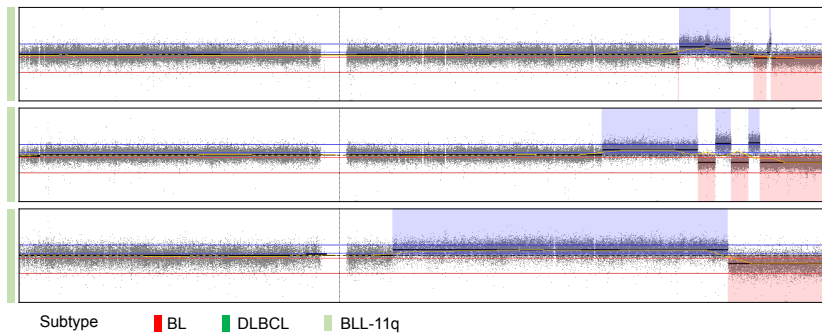

Supplement: Supplementary file 5 — Supplemental Figure 2 [file 41375_2021_1444_MOESM5_ESM.pdf]

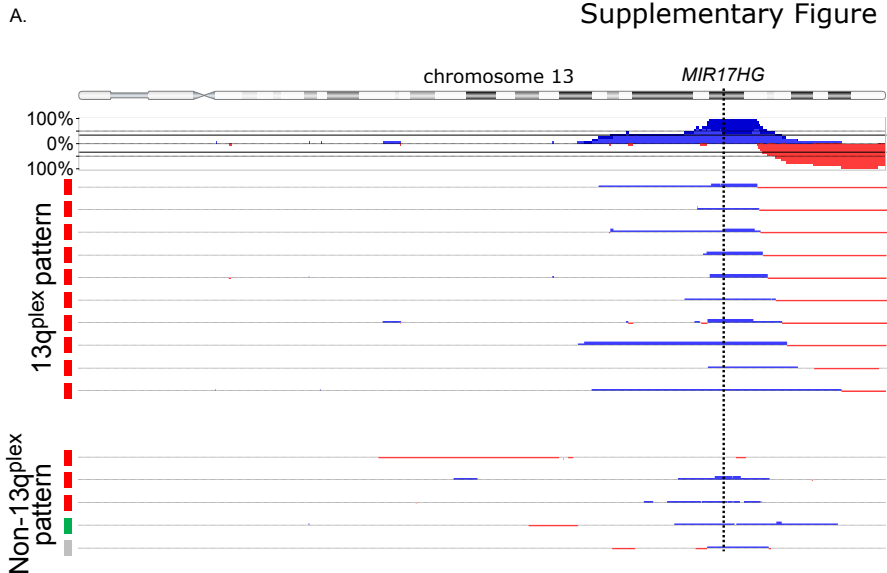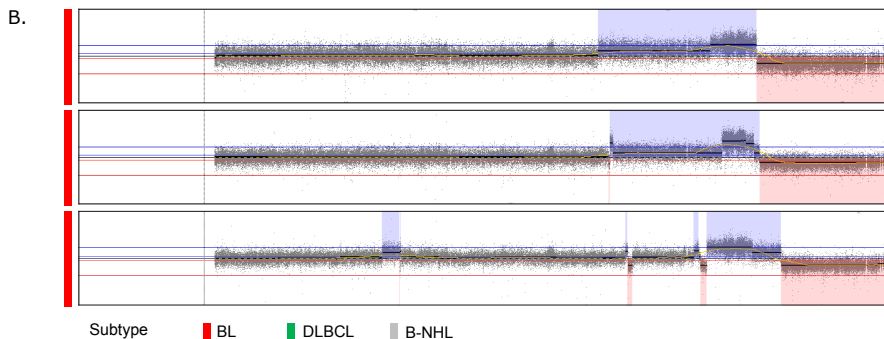

Supplement: Supplementary file 6 — Supplemental Figure 3 [file 41375_2021_1444_MOESM6_ESM.pdf]

A.

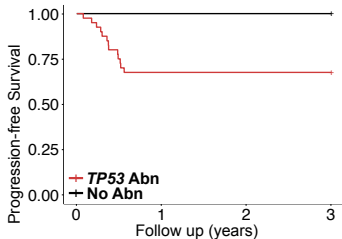

|          | Number at risk |    |    |    |
|----------|----------------|----|----|----|
| TP53 Abn | 20             | 20 | 20 | 20 |
| No Abn   | 40             | 27 | 27 | 27 |

B.

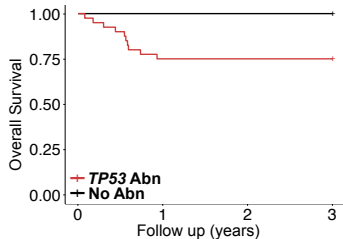

|          | Number at risk |    |    |    |
|----------|----------------|----|----|----|
| TP53 Abn | 20             | 20 | 20 | 20 |
| No Abn   | 40             | 30 | 30 | 30 |

Supplement: Supplementary file 8 — Supplemental Figure 5 [file 41375_2021_1444_MOESM8_ESM.pdf]

A.

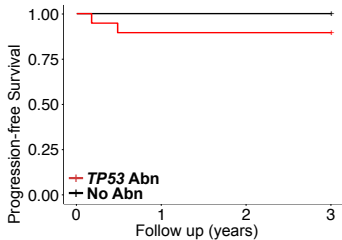

|          | Number at risk |    |    |    |
|----------|----------------|----|----|----|
| TP53 Abn | 19             | 19 | 19 | 19 |
| No Abn   | 19             | 17 | 17 | 17 |

B.

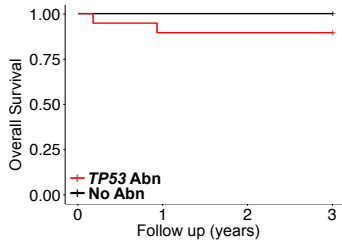

|          | Number at risk |    |    |    |
|----------|----------------|----|----|----|
| TP53 Abn | 19             | 19 | 19 | 19 |
| No Abn   | 19             | 17 | 17 | 17 |

Supplement: Supplementary file 9 — Supplemental Figure 6 [file 41375_2021_1444_MOESM9_ESM.pdf]
