# Supplementary material for: Genomic abnormalities of TP53 define distinct risk groups of paediatric B-cell non-Hodgkin lymphoma
Source: Leukemia. 2021 Oct 21;36(3):781–9. doi: 10.1038/s41375-021-01444-6 (PMC8885412; doi:10.1038/s41375-021-01444-6)
Supplement: Supplementary file 7 — Supplemental Figure 4 [file 41375_2021_1444_MOESM7_ESM.pdf]

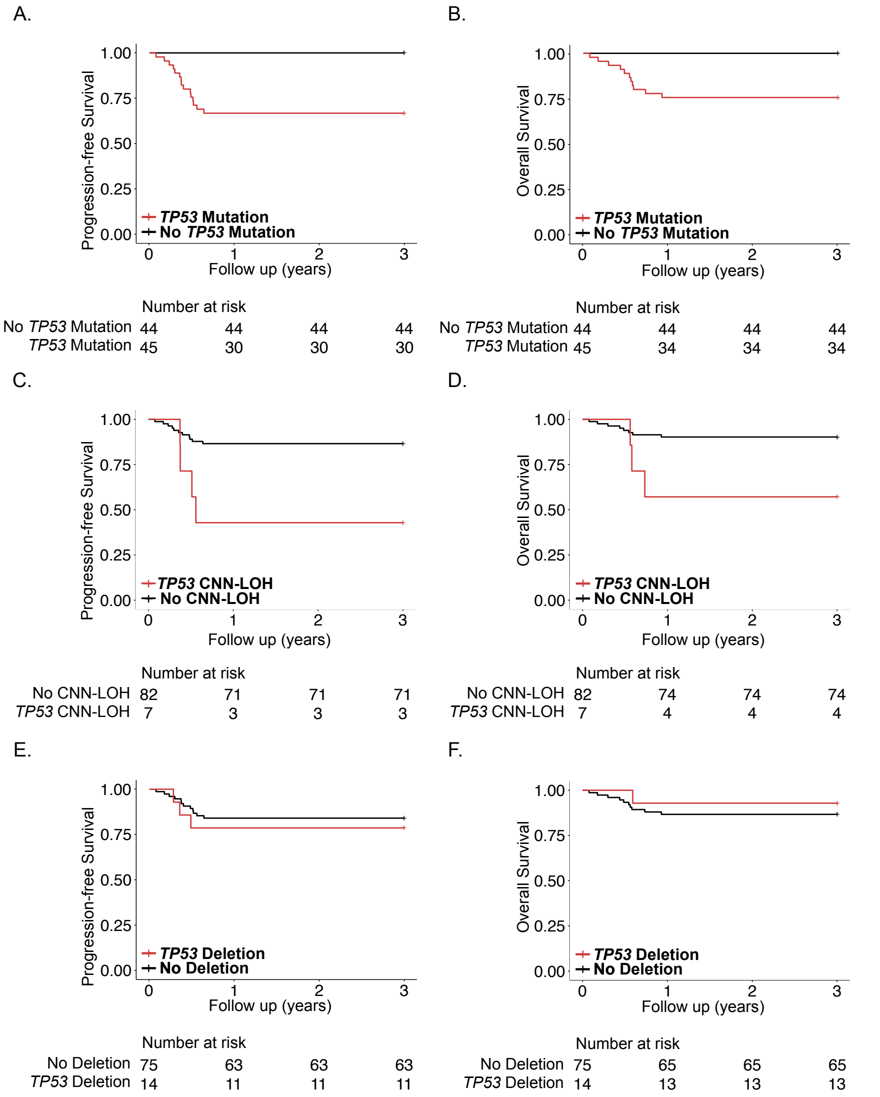
A.
Progression-free Survival
1.00
0.75
0.50
0.25
0.00
TP53 Mutation
No TP53 Mutation
0
1
2
3
Follow up (years)
Number at risk
No TP53 Mutation 44 44 44 44
TP53 Mutation 45 30 30 30
B.
Overall Survival
1.00
0.75
0.50
0.25
0.00
TP53 Mutation
No TP53 Mutation
0
1
2
3
Follow up (years)
Number at risk
No TP53 Mutation 44 44 44 44
TP53 Mutation 45 34 34 34
C.
Progression-free Survival
TP53 CNN-LOH
No CNN-LOH
Follow up (years)
Number at risk
No CNN-LOH 82 71 71 71
TP53 CNN-LOH 7 3 3 3
D.
Overall Survival
TP53 CNN-LOH
No CNN-LOH
Follow up (years)
Number at risk
No CNN-LOH 82 74 74 74
TP53 CNN-LOH 7 4 4 4
E.
Progression-free Survival
TP53 Deletion
No Deletion
Follow up (years)
Number at risk
No Deletion 75 63 63 63
TP53 Deletion 14 11 11 11
F.
Overall Survival
TP53 Deletion
No Deletion
Follow up (years)
Number at risk
No Deletion 75 65 65 65
TP53 Deletion 14 13 13 13
